# Mutations in the WG and GW motifs of the three RNA silencing suppressors of grapevine fanleaf virus alter their systemic suppression ability and affect virus infectivity

**DOI:** 10.3389/fmicb.2024.1451285

**Published:** 2024-08-12

**Authors:** Jiyeong Choi, Scottie Browning, Corinne Schmitt-Keichinger, Marc Fuchs

**Affiliations:** ^1^Plant Pathology and Plant-Microbe Biology Section, School of Integrative Plant Science College of Agriculture and Life Sciences, Cornell University, Cornell AgriTech at the New York State Agricultural Experiment Station, Geneva, NY, United States; ^2^CNRS, IBMP UPR 2357, Université de Strasbourg, Strasbourg, France; ^3^INRAE, SVQV UMR 1131, Université de Strasbourg, Colmar, France

**Keywords:** nepovirus, WG/GW, silencing suppressor, RNA silencing, virus infectivity, plant-virus interaction, AlphaFold2

## Abstract

Viral suppressors of RNA silencing (VSRs) encoded by grapevine fanleaf virus (GFLV), one of the most economically consequential viruses of grapevine (*Vitis* spp.), were recently identified. GFLV VSRs include the RNA1-encoded protein 1A and the putative helicase protein 1B^Hel^, as well as their fused form (1AB^Hel^). Key characteristics underlying the suppression function of the GFLV VSRs are unknown. In this study, we explored the role of the conserved tryptophan-glycine (WG) motif in protein 1A and glycine-tryptophan (GW) motif in protein 1B^Hel^ in their systemic RNA silencing suppression ability by co-infiltrating *Nicotiana benthamiana* 16c line plants with a *GFP* silencing construct and a wildtype or a mutant GFLV VSR. We analyzed and compared wildtype and mutant GFLV VSRs for their (i) efficiency at suppressing RNA silencing, (ii) ability to limit siRNA accumulation, (iii) modulation of the expression of six host genes involved in RNA silencing, (iv) impact on virus infectivity *in planta*, and (v) variations in predicted protein structures using molecular and biochemical assays, as well as bioinformatics tools such as AlphaFold2. Mutating W to alanine (A) in WG of proteins 1A and 1AB^Hel^ abolished their ability to induce systemic RNA silencing suppression, limit siRNA accumulation, and downregulate *NbAGO2* expression by 1AB^Hel^. This mutation in the GFLV genome resulted in a non-infectious virus. Mutating W to A in GW of proteins 1BHel and 1ABHel reduced their ability to suppress systemic RNA silencing and abolished the downregulation of *NbDCL2*, *NbDCL4,*, and *NbRDR6* expression by 1B^Hel^. This mutation in the GFLV genome delayed infection at the local level and inhibited systemic infection *in planta*. Double mutations of W to A in WG and GW of protein 1ABHel abolished its ability to induce RNA silencing suppression, limit siRNA accumulation, and downregulate *NbDCL2* and *NbRDR6* expression. Finally, *in silico* protein structure prediction indicated that a W to A substitution potentially modifies the structure and physicochemical properties of the three GFLV VSRs. Together, this study provided insights into the specific roles of WG/GW not only in GFLV VSR functions but also in GFLV biology.

## 1 Introduction

Plants utilize RNA silencing as a conserved and effective antiviral immune response against virus infections (Gaffar and Koch, 2019; Jin et al., 2022; Lopez-Gomollon and Baulcombe, 2022). This immunity is active both at the local level in the initially infected cells and their neighboring cells in a non-cell-autonomous manner, and in distant tissues (Liu and Chen, 2018; de Felippes and Waterhouse, 2020). For RNA silencing, the host dicer-like endoribonuclease (DCL) cleaves virus double-stranded RNA, intermediate products of virus replication, into virus-derived small interfering RNA (vsiRNA, a.k.a. primary siRNA) duplexes (Gaffar and Koch, 2019; Jin et al., 2022; Lopez-Gomollon and Baulcombe, 2022). One of the vsiRNA strands is then loaded onto the Argonaute (AGO) protein family of the RNA-induced silencing complex (RISC). The assembled RISC-vsiRNA targets complementary viral RNA molecules for degradation and/or inhibition of protein translation (Gaffar and Koch, 2019; Jin et al., 2022; Lopez-Gomollon and Baulcombe, 2022). This action by RISC results in cleaved viral RNA molecules that serve as template strands for the host RNA-dependent RNA polymerase 6 (RDR6) and Suppressor of Gene Silencing (SGS3) complex-mediated RNA replication, leading to the production of secondary siRNA precursors (Gaffar and Koch, 2019; Jin et al., 2022; Lopez-Gomollon and Baulcombe, 2022). The secondary siRNA molecules serve as drivers of the phloem-mediated long-distance movement of RNA silencing signals and establishment of the systemic RNA silencing (Liu and Chen, 2018; Gaffar and Koch, 2019; de Felippes and Waterhouse, 2020; Jin et al., 2022; Lopez-Gomollon and Baulcombe, 2022).

**Figure 1:**
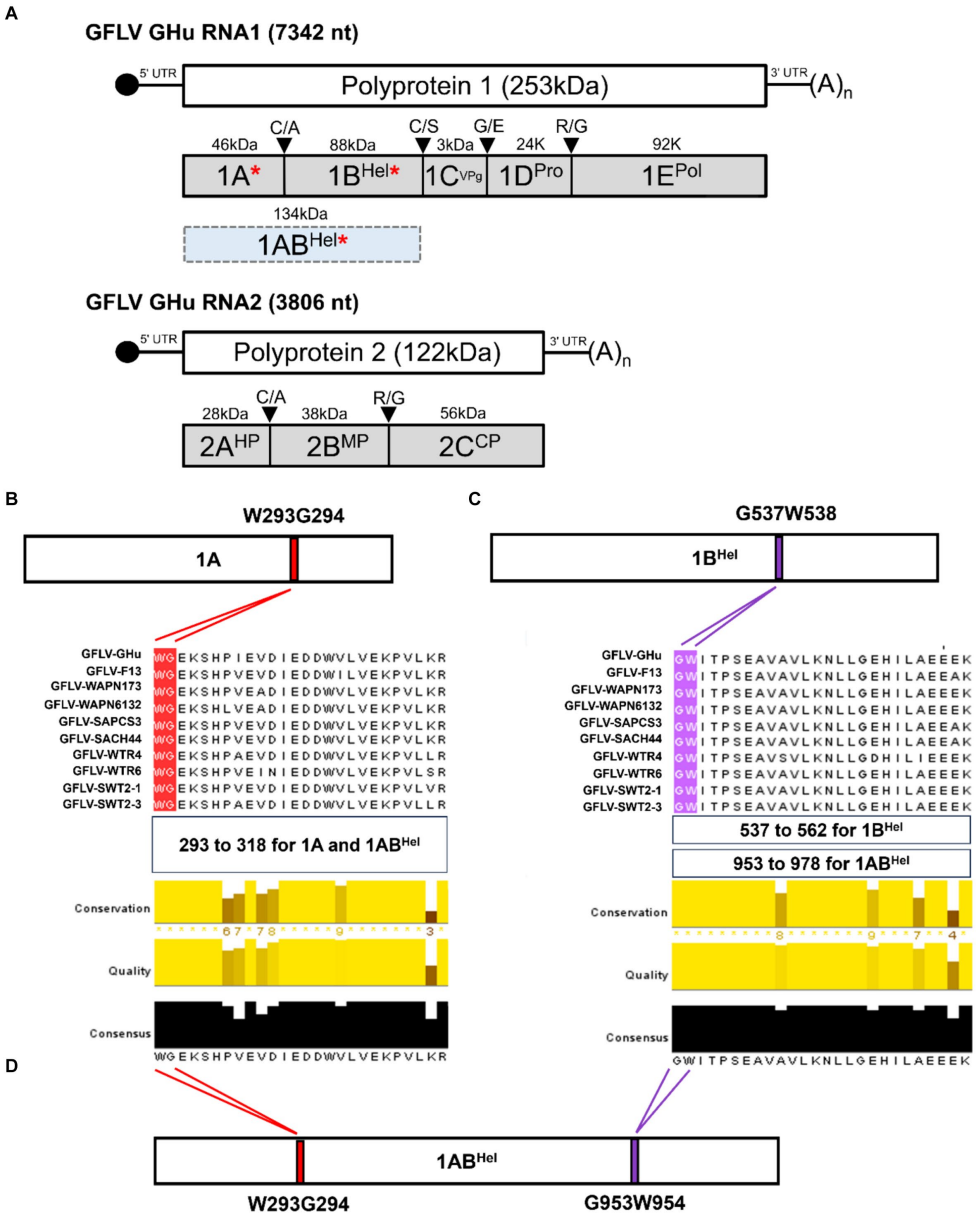
**(A)** Genome organization and proteins encoded by grapevine fanleaf virus (GFLV) strain GHu. GFLV viral RNA silencing suppressors (VSRs) 1A, 1B^Hel^, and 1AB^Hel^ are indicated by a red asterisk. Location of a WG/GW motif in GFLV P1 of several GFLV strains with panel **(B)** showing a WG motif in protein 1A [416 amino acid(aa)-long] at aa positions 293–294, and panel **(C)** showing a GW motif in protein 1B^Hel^ (801 aa-long) at aa positions 537–538. The regions of aa alignments are shown as follows: aa positions 293 to 318 for a WG motif alignment in 1A and 1AB^Hel^, aa positions 537 to 562 for a GW motif alignment in 1B, and aa positions 953 to 978 for a GW motif alignment in 1AB^Hel^. Multiple aa sequence alignments of protein 1A and 1B^Hel^ of GFLV strains GHu and F13, and eight other GFLV isolates using Jalview with conserved WG and GW motifs highlighted in red and purple, respectively. Conservation, quality, and consensus scores of the alignments are shown as histograms with the consensus aa sequences at the bottom. Maximum scores for the level of conservation, quality of alignments, and consensus sequences are shown below each alignment. **(D)** Presence of both WG and GW motifs in GFLV 1AB^Hel^ (1,217 aa-long) at aa positions 293–294 and 953–954, respectively. Images were partially generated by PyMOL v. 2.0.

Grapevine fanleaf virus (GFLV) is a member of the species *Nepovirus foliumflabelli* in the family *Secoviridae* (Fuchs et al., 2022). GFLV causes fanleaf degeneration disease, one of the most destructive viral diseases of grapevine (*Vitis* spp.) in numerous vineyards worldwide (Andret-Link et al., 2004; Schmitt-Keichinger et al., 2017; Martelli, 2019). This disease can substantially reduce fruit yield (up to 80%) and quality, and the productive lifespan of vineyards (Andret-Link et al., 2004; Schmitt-Keichinger et al., 2017; Martelli, 2019). GFLV is transmitted by the ectoparasitic dagger nematode *Xiphinema index* in a semi-persistent manner (Fuchs et al., 2017; Schmitt-Keichinger et al., 2017). The GFLV genome is composed of two positive sense single-stranded RNAs, RNA1 and RNA2, which are monocistronically translated into polyprotein 1 (P1) and polyprotein 2 (P2), respectively (Figure 1A; Fuchs et al., 2017; Schmitt-Keichinger et al., 2017). Each polyprotein is proteolytically processed by the RNA1-encoded viral cysteine protease, 1D^Pro^, through specific cleavage sites to produce mature, individual functional proteins (Figure 1A). The two GFLV genomic RNAs are necessary for systemic infection of plant hosts (Schmitt-Keichinger et al., 2017). Recently, the following three GFLV RNA1-encoded viral RNA silencing suppressors (VSRs) were identified (Figure 1A): proteins 1A (46 kDa), 1B^Hel^ (88 kDa), and 1AB^Hel^ (134 kDa), a fusion protein of 1A and 1B^Hel^, which was predicted as an intermediary product of 1D^Pro^-mediated *cis*-proteolytic processing (Choi et al., 2023). GFLV VSRs reverse systemic RNA silencing and differentially alter the expression of host genes involved in RNA silencing (Choi et al., 2023). For simplicity, the three GFLV VSRs will be referred to as proteins 1A, 1B, and 1AB in the remainder of the manuscript.

A tryptophan-glycine (WG) or a glycine-tryptophan (GW) is a protein sequence feature known as a WG/GW motif (Eystathioy et al., 2002; El-Shami et al., 2007). This motif is found in some plant VSRs and is critical for their suppression functions through interaction with AGO and siRNA (Giner et al., 2010; Csorba et al., 2015; Pérez-Cañamás and Hernández, 2015; Li et al., 2018; Pollari et al., 2020). Mutating the WG/GW motif abolishes or reduces the suppression activity in such VSRs, including but not limited to protein P1 of sweet potato mild mottle virus (SPMMV; genus *Ipomovirus*, family *Potyviridae*; Giner et al., 2010), p24 of grapevine leafroll-associated virus 2 (GLRaV 2; *genus Closterovirus*, family *Closteroviridae*; Li et al., 2018), and p37 of *Pelargonium* line pattern virus (PLPV; genus *Pelarspovirus*, family *Tombusviridae*; Pérez-Cañamás and Hernández, 2015). Similarly, the role of WG/GW in the VSR function of a nepovirus was demonstrated for tomato ringspot virus (ToRSV; Karran and Sanfaçon, 2014). Mutating W to alanine (A) in WG located near the C-terminus of the ToRSV coat protein (CP) coding region abolishes its binding to AGO1 protein, thus inhibiting its ability to suppress RNA silencing (Karran and Sanfaçon, 2014). For P1 of SPMMV, p24 of GLRaV 2, and p37 of PLPV, single or double mutations in WG/GW located at the N-terminus region of the VSRs abolish RNA silencing suppression (Giner et al., 2010; Pérez-Cañamás and Hernández, 2015).

In this study, we screened the amino acid sequence of the polyprotein encoded by GFLV RNA1 for a WG/GW motif and examined the role of W in systemic RNA silencing suppression function through mutagenesis and co-infiltration experiments in transgenic *Nicotiana benthamina* 16c plants constitutively expressing the green fluorescent protein (GFP). Here we report our findings on the critical role of W in the WG/GW motif of GFLV VSRs for suppressing systemic RNA silencing, reducing siRNA accumulation, and differentially regulating the expression of host genes involved in RNA silencing, as well as in establishing systemic infection.

## 2 Materials and methods

### 2.1 *In silico* characterization of the WG/GW motif in polyprotein P1 sequences of nepoviruses and GFLV VSRs

The polyprotein P1 amino acid sequences of GFLV strains GHu (GFLV-GHu) and F13 (GFLV-13) and eight other GFLV strains were retrieved from GenBank and analyzed for the presence of WG or GW motifs using DNASTAR Lasergene v. 17 (Supplementary Table 2). The eight GFLV strains were randomly selected from those with annotated P1 sequences available in GenBank to assess their divergence from GFLV strains GHu and F13. Multiple amino acid sequence alignments were conducted using Clustal Omega with ClustalW (Sievers and Higgins, 2018). Jalview software (v. 2.11.2.6) was used to align P1 sequences of GFLV isolates and to calculate conservation, consensus, and quality scores of the alignments (Clamp et al., 2004). Similarly, the P1 sequences of several other nepoviruses were analyzed for the presence of WG and GW, and their locations were compared with those identified in GFLV VSRs.

AlphaFold2 ColabFold v1.5.5 with MMseqs2 (accessed on 2.27.2024; Steinegger and Söding, 2017; Mirdita et al., 2022) and RoseTTAFold (accessed on 2.29.2024; Baek et al., 2021) were used to predict the structure of the VSRs of GFLV strains GHu and F13 (GFLV-F13). Similarly, these programs were utilized to explore the protein structure of wildtype and mutant GFLV-GHu VSRs. The mutant GFLV-GHu VSR protein sequences were manually edited (W to A) and analyzed. The confidence scores for each protein structure prediction were recorded. The visualization and characterization of predicted structures were carried out with PyMOL v. 2.0 (Schrödinger and DeLano, 2020) and ChimeraX (Goddard et al., 2018; Pettersen et al., 2021; Meng et al., 2023). Template modeling (TM)-align was used to conduct pairwise comparisons of protein structures between wildtype and mutant GFLV VSRs based on a sequence-independent approach (accessed on 2.29.2024; Zhang and Skolnick, 2005). The outcome of TM-align was displayed in TM-score format. TM-score is a scale system evaluating the similarity of protein structures with a maximum score of 1 (indicating complete similarity) and a minimum score of 0 (Zhang and Skolnick, 2004; Xu and Zhang, 2010). A TM-score above 0.5 indicates that the fold of two proteins is relatively similar, while a TM-score below 0.2 indicates that two protein structures are randomly associated with each other based on SCOP and CATH methods (Zhang and Skolnick, 2004; Csaba et al., 2009; Xu and Zhang, 2010). In this study, we determined the TM-score between the most top-ranked models with highest accuracy in structure prediction for AlphaFold2 and top one- and two-ranked models for RoseTTAFold.

### 2.2 GFLV-GHu VSR mutant constructs

**Figure 2:**
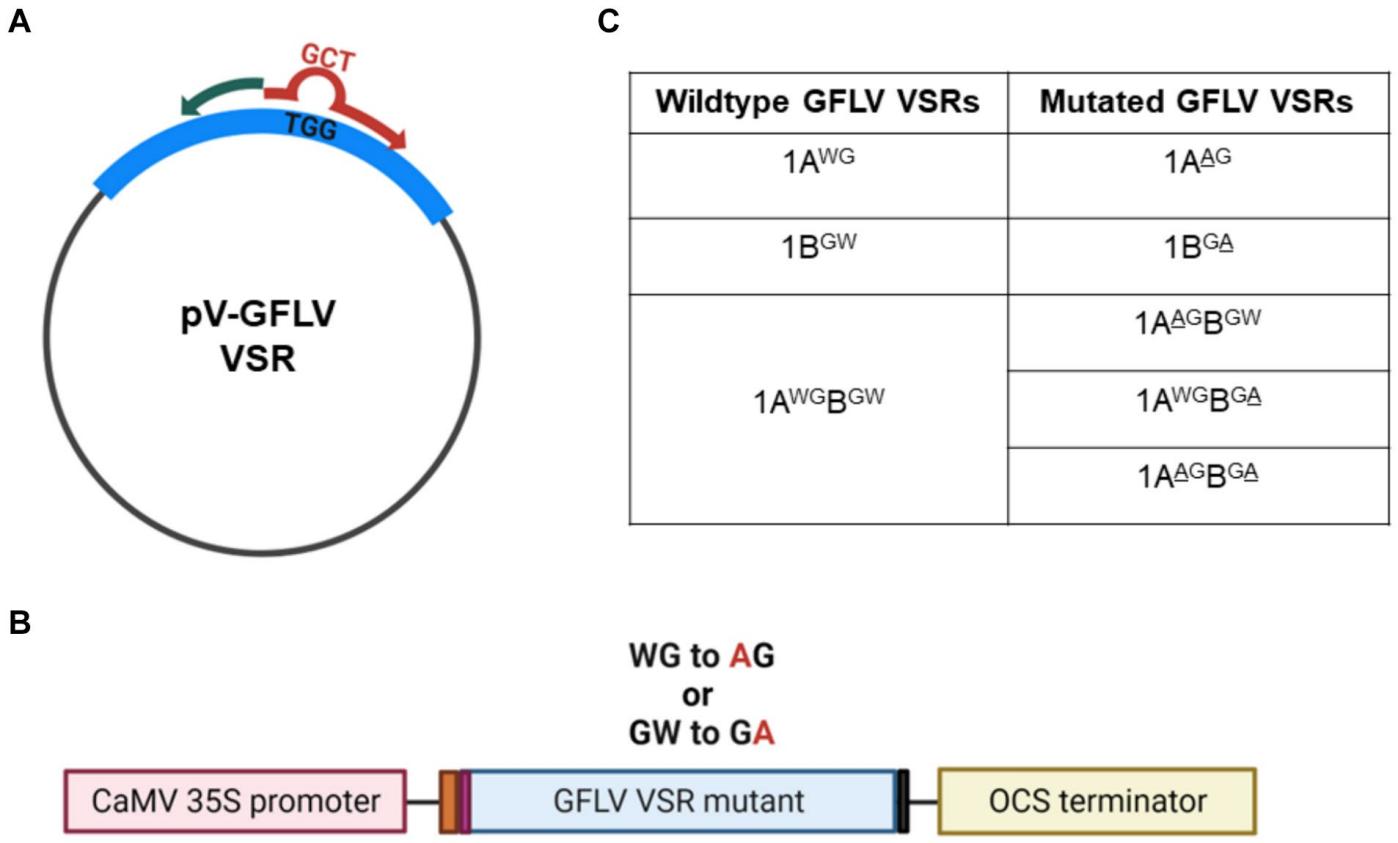
Site-directed mutagenesis of grapevine fanleaf virus (GFLV) viral RNA silencing suppressors (VSRs). **(A)** Substitution mutations were carried out with pEarleyGate100 (pV) encoding wildtype GFLV VSRs as a template. The mutagenic forward primer (in red) contains the desired nucleotide sequences (GCT) encoding alanine (A) to replace the original nucleotide sequence (TGG) encoding tryptophan (W) of wildtype GFLV VSRs. The reverse primer (in green) is complementary to the template. **(B)** Diagram of GFLV VSR mutant expression cassette composed of, from left to right, the cauliflower mosaic virus (CaMV) 35S promoter (pink box), the Kozak sequence (CCAAC; orange box), a start codon encoding methionine (Met; purple box), a wildtype or a mutant GFLV VSR sequence, a stop codon (gray box), and an octopine synthase (OCS) terminator sequence (yellow box). Met is the original starting residue for 1A and 1AB, and a Met residue was incorporated for 1B using the *att*B1 Gateway cloning primer (Choi et al., 2023). **(C)** List of GFLV VSR mutants created by site-directed mutagenesis with the mutated amino acid underlined.

Binary constructs of mutant GFLV-GHu VSRs were created using pEarleyGate100 (pV)-GFLV-1A, -1B, and -1AB as templates (Earley et al., 2006; Choi et al., 2023). The empty plasmid pV contains a cauliflower mosaic virus (CaMV) 35S promoter and the 3′-flanking region of the octopine synthase gene (OCS; Figure 2A). A Kozak sequence (CCAAC) was inserted upstream of the GFLV VSR sequence for optimal ribosomal binding. The Q5^®^ Site-Directed Mutagenesis Kit (New England Biolabs) was used to create substitution mutations with specific primers (Supplementary Table 1) designed by the NEBaseChanger® software v 2.0.0. The mutagenic primers were complementary to the target GFLV sequences, except three nucleotides in the forward primer that were designed to replace TGG (W) with GCT (A, alanine). The most preferred codon (GCT) for A in *N*. *benthamiana* and *V. vinifera* was chosen to create GFLV-GHu VSR mutant constructs to ensure codon optimization (Atef et al., 2020; Sadovskaya et al., 2021). Single or double substitution mutations from W to A in the WG or GW motifs of GFLV 1A, 1B, or 1AB were verified via Sanger sequencing at the Cornell Biotechnology Resource Center in Ithaca, New York. Nucleotide and resulting amino acid sequences were analyzed with DNASTAR Lasergene v. 17. The resulting mutant constructs were transformed into competent *A. tumefaciens* strain GV3101 cells via electroporation. Colony PCR followed by Sanger sequencing was conducted to screen and identify a single isolated colony of *A. tumefaciens* GV3101 containing the desired mutant GFLV VSR construct. The resulting mutant constructs of GFLV 1A, 1B, and 1AB proteins are referred to as 1A^AG^, 1B^GA^, 1A^AG^B, 1AB^GA^, and 1A^AG^B^GA^. Mutant construct 1A^AG^B has a WG to AG swap at positions 293–294 of 1AB, while mutant construct 1AB^GA^ has a GW to GA swap at positions 953–954 of 1AB. Mutant construct 1AAGBGA has both WG to AG and GW to GA swaps.

### 2.3 Plant material

Transgenic *N*. *benthamiana* plants expressing GFP (line 16c; Voinnet and Baulcombe, 1997) were used to characterize wildtype and mutant GFLV-GHu VSRs. Plants were grown and maintained in a walk-in growth chamber at 25°C with a 16 h light and 8 h dark cycle with 50% humidity. Wildtype *Chenopodium quinoa* plants were grown and assessed for GFLV-F13 infection under similar growth chamber condition used for *N*. *benthamiana* plants.

### 2.4 GFLV-F13 recombinant constructs and infection of plants with transcripts

Sequences coding for the fluorescent protein Venus (Nagai et al., 2002) or TagRFP (Merzlyak et al., 2007) were introduced into the infectious clone pMV13 corresponding to RNA1 of GFLV strain F13 (Viry et al., 1993) via overlap PCR mutagenesis. First, an *Avr*II restriction site was introduced into pMV13 at the junction between the 5’-UTR and the 1A coding sequence of pMV13 (Viry et al., 1993). Then, primers 353LR1ST7 and 29AvrNT1AR were used for overlap PCR to amplify a fragment ranging from the *Sal*I site upstream of the T7 transcription promoter to 798 nucleotides downstream of the *Age*I site in the 1A coding sequence (Supplementary Table 1). Next, the resulting overlap PCR fragment was subcloned into pMV13 at *Sal*I and *Age*I sites, resulting in the pMV13-Avr21 clone. The Venus and TagRFP coding sequences were amplified from plasmids pSiteII-4C1 (Martin et al., 2009) with primers 297AvrVenusF and 298AvrVenusR and from plasmid pTagRFP (Evrogen) with primers 132AvrTagRFPF and 133AvrTagRFPR (Supplementary Table 1). The Venus and TagRFP fragments were subcloned into pMV13-Avr21 at the *AvrII* site to generate recombinant GFLV-F13 RNA1 infectious clone fused with Venus (pMV13-Ve1A) or TagRFP (pMV13-TR1A) at the N-terminus of 1A (Schmitt-Keichinger et al., 2017).

Site-directed mutagenesis PCR was conducted to substitute W with A in positions 293 and 954 in pMV13 using a phosphorylated mutagenic oligonucleotide in pair with a non-mutagenic primer. The residue W of 1A at position 293 was substituted with A (GCC), and the residue G (GCC) of 1A of pMV13 at position 294 was substituted with a different codon (GGG) with primers 2831AW293AF and 2841Aseq876R (Supplementary Table 1). The forementioned codons were chosen to introduce a *Nae*I restriction site to facilitate cloning. The mutagenesis PCR replaced residue W of 1B of pMV13 at position 954 with A (GCG) using primers 4981BW854A and 4991Bseq3100R. The forementioned codon was chosen for the optimization of primer pair efficiency (Supplementary Table 1). GFLV-F13 RNA1 fragments carrying the mutations were subcloned into pMV13-Ve1A and pMV13-TR1A via restriction digestion at *Age*I-*Xba*I and *Apa*I followed by ligation to generate pMV13-Ve1A^AG^ and pMV13-TR1A^AG^ or pMV13-Ve1AB^GA^ and pMV13-TR1AB^GA^, respectively. The mutations in recombinant pMV13 were verified by Sanger sequencing.

Infectious recombinant transcripts of GFLV-F13 RNA1 and those with mutations (1A^AG^ or 1AB^GA^) were produced using the mMessage mMachine® T7 kit (Ambion) after linearization of recombinant pMV13 with *Bgl*II (Martin et al., 2018). From the resulting products, the following infectious transcripts of GFLV-F13 RNA1 were used in the virus infection experiments: RNA1 Venus-1A, RNA1 TagRFP-1A, RNA1 Venus-1A^AG^, RNA1 Venus-1AB^GA^, and RNA1 TagRFP-1AB^GA^. A similar approach was used to produce infectious transcripts of recombinant GFLV-F13 RNA2 tagged with TagRFP (RNA2 2A-TagRFP) or EGFP (RNA2 2A-EGFP; Zhang et al., 1996) from pVecP2 after *Sal*I linearization (Schmitt-Keichinger et al., 2017; Martin et al., 2018). Viral transcripts were recovered using lithium chloride precipitation followed by resuspension in RNase free water. The size and integrity of the GFLV-F13 RNA1 and RNA2 transcripts were verified by denaturing agarose gel electrophoresis.

Infectious recombinant GFLV-F13 RNA1 transcripts, including those with 1A or 1B mutations, and recombinant RNA2 transcripts were mechanically inoculated onto *C. quinoa*, as previously described (Schmitt-Keichinger et al., 2017; Martin et al., 2018). Transcripts of RNA1 tagged with Venus (RNA1 Venus-1A, RNA1 Venus-1A^AG^, or RNA1 Venus-1AB^GA^) were co-inoculated with transcripts of RNA2 2A-TagRFP. Similarly, transcripts of RNA1 tagged with TagRFP (RNA1 TagRFP-1A or RNA1 TagRFP-1AB^GA^) and RNA2 2A-EGFP were co-inoculated. Two leaves per plant were inoculated for each treatment, and 10 plants were used in three independent experiments. Infection was assessed in inoculated leaves and in uninoculated, apical leaves of *C. quinoa* plants by fluorescence observation using an Axiozoom V16 stereomicroscope (Zeiss). The excitation and emission filter sets of 625–655 nm and 665–715 nm; 450–490 nm and 500–550 nm; and 538–562 nm and 570–640 nm were used for the visualization of chlorophyll, EGFP or Venus and TagRFP, respectively.

### 2.5 Co-infiltration of transgenic *N. benthamiana* 16c plants with an RNA silencing inducer and wildtype and mutant GFLV-GHu VSR constructs

*Agrobacterium tumefaciens* strain GV3101 cells containing pV-1A, pV-1A^AG^, pV-1B, pV-1B^GA^, pV-1AB, pV-1A^AG^B, pV-1AB^GA^, pV-1A^AG^B^GA^ and empty vector pV were grown in Luria-Bertani liquid media supplemented with 100 μM of acetosyringone and appropriate antibiotics (gentamicin 30 mg/L, kanamycin 50 mg/L, and rifampicin 15 mg/L) at 28°C with shaking at 225 rpm for no more than 2 days. Concurrently, *A. tumefaciens* GV3101 cells harboring the RNA silencing-inducing hairpin construct pHELLSGATE8-EGFP (Helliwell and Waterhouse, 2003; Choi et al., 2023; gentamicin 30 mg/L, rifampicin 15 mg/L, spectinomycin 100 mg/L) and *A. tumefaciens* strain C58Z707 cells harboring pGA482G-GLRaV2 p24 (Osterbaan et al., 2018; gentamicin 30 mg/L and kanamycin 50 mg/L) were similarly grown. The bacterial cultures were then diluted in the infiltration buffer (10 mM MES-KOH pH 5.6, 10 mM MgCl_2_, and 200 μM acetosyringone; Choi et al., 2023).

Bacteria used in co-infiltration experiments were prepared, as previously described (Choi et al., 2023) with slight modifications. Briefly, agrobacteria harboring pHELLSGATE8-EGFP (OD_600nm_ of 1.0) were mixed at a 1:1 ratio (v:v) with agrobacteria harboring a wildtype or a mutant GFLV VSR construct (OD_600nm_ of 0.4–5) prior to infiltration. The same mixing of agrobacteria was also applied for negative control pV and positive control p24 of GLRaV2 (Earley et al., 2006; Li et al., 2018; Choi et al., 2023). Sterile 3 mL syringes without needles were used to introduce agrobacterial cultures through the abaxial surfaces of two to three youngest leaves of the four to seven leaf development stage plants. Plants infiltrated with buffer or solely agrobacteria harboring pHELLSGATE8-EGFP were used as negative controls. Agroinfiltration assays were repeated three times with three to seven biological replicates (plants) per treatment.

### 2.6 Monitoring systemic RNA silencing suppression and tissue collection

Transgenic *N. benthamiana* 16c plants expressing *GFP* (Voinnet and Baulcombe, 1997) were monitored daily under a hand-held high-intensity UV lamp for GFP fluorescence (UVP Black-Ray® B100APR, Analytik Jena) starting at 2 days post-infiltration (dpi). Fluorescence photos were taken using a digital camera NIKON D850 with no filter and light gathering setting at 1,000. For visualizing the systemic movement of RNA silencing of *GFP* in *N*. *benthamiana* 16c plants, both the stems and apical leaves of each biological replicate (plant) were observed under UV light starting at 2 dpi.

Leaf tissues were collected from the second or third youngest apical leaf of each biological replicate at approximately 20 dpi for analysis of silencing suppression. Eight leaf disc samples were collected per biological replicate using a no. 4 corkborer. Two leaf discs were used for GFP spectrometry, analyses of host gene expressions by RT-qPCR, and siRNA quantification analyses by stem-loop RT-qPCR (Varkonyi-Gasic and Hellens, 2011; Tarquini et al., 2021). The remaining two leaf discs were saved as backups.

### 2.7 GFP expression analyses

Co-infiltrated plants were monitored for systemic *GFP* silencing starting at 2 dpi and leaf samples were collected for analyses around 20 dpi. GFP fluorescence intensity was measured by spectrometry using two leaf discs per biological replicate, as previously described (Choi et al., 2023). First, leaf discs were homogenized at 30 Hz for 2 min with a MM400 mixer mill (Retsch). Then, lysates were suspended in extraction buffer (50 mM Tris–HCl pH 8.0, 150 mM NaCl, and 10 mM EDTA) followed by centrifugation at 12,000 *g* for 15 min at 4°C. Next, total soluble proteins in the supernatant were loaded in duplicate in clear 96-well microtiter plates (Thermo Scientific™) and screened for GFP fluorescence intensity using a Synergy2 microplate reader (BioTek) with specific excitation at 360 nm with a bandpass of 40 and emission at 508 nm with a bandpass of 20.

The relative accumulation of GFP protein was measured via SDS-PAGE followed by western blot with specific GFP antibodies using total proteins extracted from combined leaf tissues of three biological replicates of the same treatment with lysis buffer (50 mM HEPES-KOH pH 7.4, 110 mM KOAc, 2 mM MgCl_2_, 0.4% TritonX-100, 2.5 mM DTT, and 1X Protease Inhibitor Cocktail from Invitrogen; Choi et al., 2023).

### 2.8 Expression analyses of host genes involved in RNA silencing by RT-PCR

Two leaf discs per biological replicate were used to extract total RNA using the E.Z.N.A. Plant RNA kit (Omega Bio-Tek) as per the manufacturer’s instructions including DNase-I treatment. Total RNAs exhibiting an A260nm/A280nm ratio below 2.1 were excluded from further analyses. The SYBR-green based Luna® Universal One-Step RT-qPCR Kit (New England Biolabs) was used with specific primers (Supplementary Table 1) on a CFX96 Touch thermocycler (Bio-Rad) to measure the relative expression of the RNA silencing-associated host genes: *NbAGO1*, *NbAGO2*, *NbDCL2*, *NbDCL4*, *NbDRB4*, and *NbRDR6*. DCL-DRB complex cleaves viral dsRNAs into vsiRNAs, in which one of the strands loads onto AGO, forming the core component of RISC. The RISC-cleaved viral ssRNAs are converted into dsRNAs by RDR6-SGS3 complex. These dsRNAs serve as precursors for secondary siRNAs, thereby amplifying the RNA silencing response. Three technical replicates were used per biological replicate (plant). The RT-qPCR quantification cycle values were recorded and normalized to the housekeeping gene *F-BOX* (Liu et al., 2012; Choi et al., 2023) and calibrated to untreated transgenic *N. benthamiana* 16c plants. The relative expression of target genes was calculated via the 2^–∆∆Ct^ method (Livak and Schmittgen, 2001).

### 2.9 Systemic siRNA quantification by stem-loop RT-qPCR

Two leaf discs per biological replicate were used to extract small RNAs using *mir*Vana (Invitrogen) as per manufacture’s protocol with slight modifications. Briefly, following the tissue lysing step, plant tissue in lysis buffer (1,3 ratio of w,v) and the phenol mixture were centrifuged at 12,000 *g* for 10 min at 4°C. The supernatant from the upper aqueous phase was used for small RNA (sRNA) extraction. The concentration of sRNAs was quantified using the Qubit™ fluorometer (2.0) with RNA assay kits (Invitrogen), and the quality of sRNAs was assessed using NanoDrop One (Thermo Scientific). Small RNAs exhibiting an A260nm/A280nm ratio below 1.5 were excluded from further analyses.

The integrity of sRNAs was analyzed by electrophoresis on denaturing 15% Mini-PROTEAN® TBE-Urea Gel (Bio-Rad) in Gel Loading Buffer II (Invitrogen) along with specific small RNA ladder (Low Range ssRNA Ladder, New England BioLabs). Following electrophoresis, ribonucleic acids were stained with SYBR™ Gold Nucleic Acid Gel Stain (Invitrogen) and visualized with a UV transilluminator.

The quantified sRNAs were used in stem-loop RT-qPCR-mediated siRNA quantification as previously described (Varkonyi-Gasic and Hellens, 2011; Tarquini et al., 2021) with slight modifications. First, 10 ng of small RNAs and 2 pmol of gene-specific stem-loop primers (Supplementary Table 1) were mixed and incubated for 5 min at 70°C. Then, reagents from the SuperScript III Reverse Transcriptase (Invitrogen) kit and 40 units of RNaseOUT™ Recombinant RNase Inhibitor (Invitrogen) were added and incubated for 30 min at 16°C, and pulsed reverse transcription was performed with 60 cycles at 30°C for 30 s, 42°C for 30 s, and 50°C for 1 s (Varkonyi-Gasic and Hellens, 2011; Tarquini et al., 2021), followed by reverse transcriptase inactivation at 85°C for 5 min. A GFP-stem-loop primer was used for GFP siRNA cDNA synthesis (Supplementary Table 1; Tarquini et al., 2021), while a noncoding small nuclear RNA U6-targeting stem-loop primer was used for reference *U6* siRNA cDNA synthesis (Supplementary Table 2; Turner et al., 2013). The resulting cDNA (2 μL) from the reverse transcription step was used for qPCR with gene-specific forward primers and universal reverse primer (Supplementary Table 2; Varkonyi-Gasic et al., 2007) using iTaq Universal SYBR Green Supermix (Bio-Rad) at 95°C for 3 min followed by 39 cycles of 95°C for 5 s, 60°C for 15 s, and 72°C for 1 s on a CFX96 Touch thermocycler (Bio-Rad). Three technical replicates were used per biological replicate, and the 2^–∆∆Ct^ method was used to quantify the relative abundance of siRNA (Livak and Schmittgen, 2001) with normalization to the reference nuclear siRNA *U6* (Turner et al., 2013) and calibration to untreated transgenic *N. benthamiana* 16c plants.

### 2.10 Statistical analyses

Quantitative data were analyzed for statistical significance using the software R (R Core Team, 2023). The normality of the sample mean distribution was evaluated via Quantile-Quantile plot and histogram. The density histogram was used to analyze overall distribution of data sets. The Kruskal–Wallis test followed by pairwise Wilcoxon test was used for unequally distributed data. For normally distributed data, one-way ANOVA followed by Dunnett’s *post hoc* multiple comparison test was used for data sets with normality. Normally distributed data with skewness were log transformed first and then analyzed using one-way ANOVA followed by Dunnett’s *post hoc* multiple comparison test.

## 3 Results

### 3.1 GFLV 1A and 1B encode conserved WG and GW motifs, respectively

*In silico* analyses revealed a WG motif at amino acid (aa) positions 293–294 of GFLV protein 1A (416 aa-long), and a GW motif at aa positions 537–538 of GFLV protein 1B (801 aa-long; Figures 1A,B). These two motifs are conserved among all GFLV isolates analyzed in this study, as shown by multiple sequence alignments (Figures 1A,B). As expected, GFLV 1AB (1,217 aa-long), the fused product of GFLV proteins 1A and 1B, encodes both WG and GW motifs at aa positions 293–294 and 953–954, respectively (Figure 1C). Maximum scores for the level of conservation (similarity in physiochemical properties), quality of alignments (proportion of the most frequent residues), and consensus sequences (inverse of mutation likelihood) were obtained for both WG and GW across all 10 GFLV isolates tested in this study (Figure 1).

At least one WG motif and one GW motif was identified in nepovirus RNA1-encoded polyprotein P1 aa sequences retrieved from GenBank (Supplementary Tables 2, 3; Supplementary Figure 1). For each nepovirus, a complete P1 coding sequence was chosen as a reference sequence. The GenBank accession numbers for these reference sequences are provided in Supplementary Tables 2, 3. A WG motif was conserved in the P1 sequence of GFLV strains GHu and F13, arabis mosaic virus (ArMV), and grapevine deformation virus (GDefV) at aa positions 293–294, while a GW motif was conserved in the P1 sequence of GFLV strains F13 and GHu, ArMV, GDefV, grapevine nepovirus A, tobacco ringspot virus, Aeonium ringspot virus, and potato black ringspot virus at aa positions 953–954 (Supplementary Table 3; Supplementary Figure 1). Other nepoviruses showed a WG/GW motif at different locations of the P1 sequence (Supplementary Table 3).

Based on the presence of conserved WG and GW motifs across multiple GFLV strains, we hypothesized that these motifs play critical roles in GFLV VSR function. To test this hypothesis, we created 1A, 1B, and 1AB mutants with a W to A substitution in WG and/or GW of GFLV-GHu, resulting in the following mutant VSRs: 1A^AG^, 1B^GA^, 1A^AG^B, 1AB^GA^, and 1A^AG^B^GA^ (Figure 2).

### 3.2 Mutating the WG motif in GFLV 1A and 1AB abolishes their systemic RNA silencing suppression ability, while mutating the GW motif in 1B and 1AB reduces their systemic RNA suppression ability

To explore the role of the WG/GW motifs in GFLV VSR functions, wildtype (1A, 1B, and 1AB) and mutant (1A^AG^, 1B^GA^, 1A^AG^B, 1AB^GA^, and 1A^AG^B^GA^) VSRs of GFLV-GHu were introduced in binary expression vectors (Figure 2) for co-infiltration experiments with pHELL and either wildtype or mutant VSR in transgenic *N. benthamiana* line 16c plants. For this study, plants with at least four apical leaves with complete restoration of *GFP* expression were categorized as exhibiting suppression of systemic RNA silencing.

**Figure 3:**
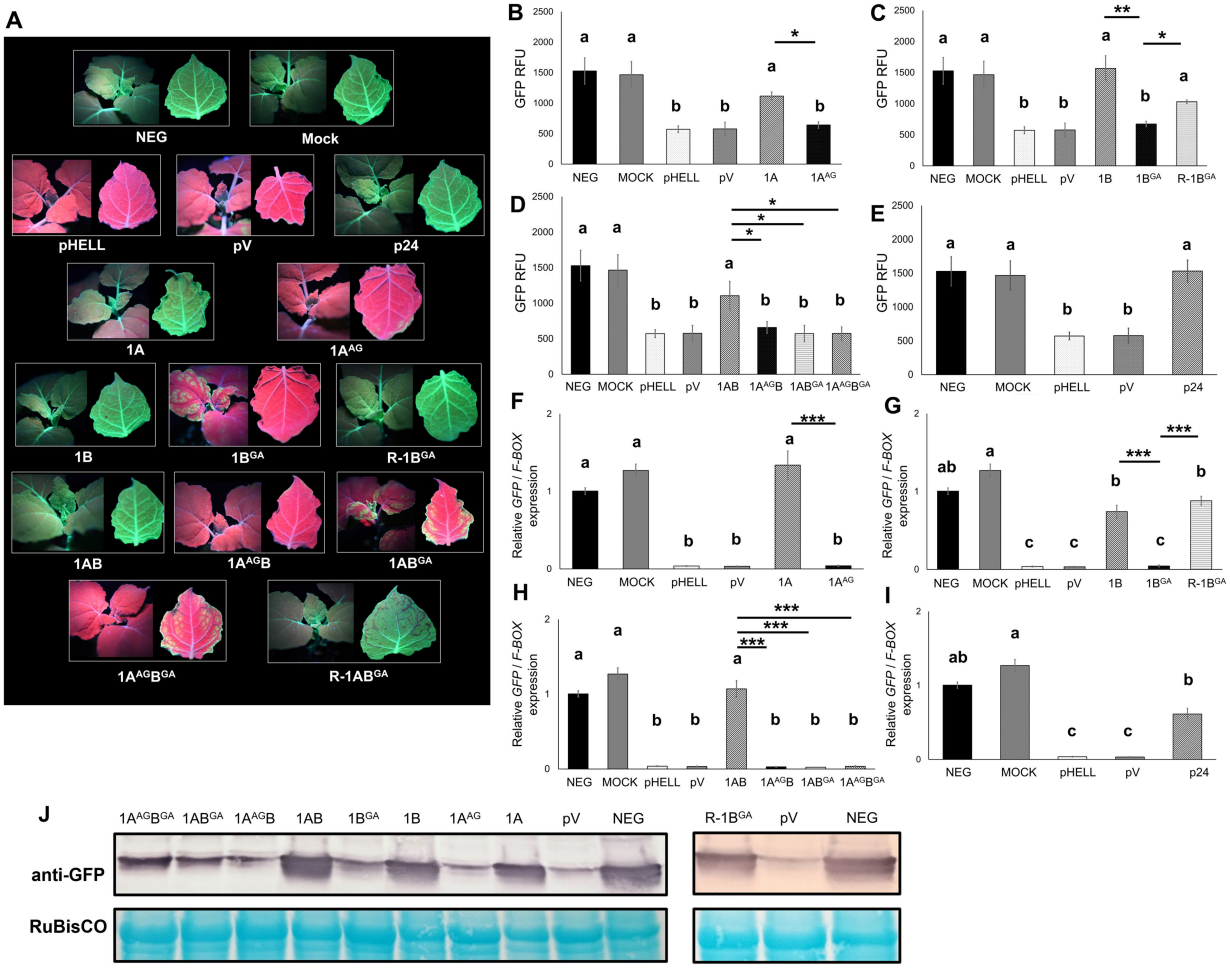
Effects of mutations in the WG/GW motif of viral RNA silencing suppressors (VSRs) encoded by grapevine fanleaf virus (GFLV) on systemic RNA silencing suppression at approximately 20 days post-infiltration. **(A)** Fluorescence images of *Nicotiana benthamiana* 16c line plants co-infiltrated with the RNA silencing inducing construct pHELLSGATE8-EGFP (pHELL) and one of the wildtype (1A, 1B, and 1AB) or mutant (1A^AG^, 1B^GA^, 1A^AG^B, 1AB^GA^, and 1A^AG^B^GA^) GFLV VSRs. For each image, the left panel exhibits the top of the plant, while the right panel exhibits the abaxial surface of the apical leaf. Photos were taken under a high-intensity UV lamp using a NIKON 850 digital camera. In the first row, an untreated 16c plant used as control is labeled as negative, and a mock-infiltrated 16c plant is labeled as mock. In the second row, pHELL and empty vector pEarleyGate100 (pV) were used as RNA silencing controls, and protein p24 (p24) of grapevine leafroll-associated virus 2 was used as a positive control. In the third row, wildtype 1A suppresses systemic RNA silencing (left), while mutant 1A^AG^ does not (right). In the fourth row, wildtype 1B suppresses systemic RNA silencing (left), while mutant 1B^GA^ either fails (center) or suppresses (R-1B^GA^; right) systemic RNA silencing. The R (reduced systemic RNA silencing suppression ability) phenotype was observed in 21% (4/19 plants) of the plants treated with 1B^GA^. In the fifth row, wildtype 1AB suppresses systemic RNA silencing (left), while the single mutants 1A^AG^B or 1AB^GA^ do not. In the sixth row, the double mutant 1A^AG^B^GA^ inhibits systemic RNA silencing (left), while 1AB^GA^ occasionally suppresses systemic RNA silencing (R-1AB^GA^; right). The R phenotype was observed in 17% (3/18 plants) of the plants treated with R-1AB^GA^. **(B–E)** Fluorescence intensity of GFP measured in relative fluorescence unit (RFU). Two technical replicates were used per biological replicate (plant). **(F–I)** Relative *GFP* expression normalized against the housekeeping gene *F-BOX* and calibrated to untreated 16c plants. Three technical replicates were used per biological replicate (plant). The asterisks denote significant differences (*p* < 0.05*, *p* < 0.01**, and *p* < 0.001***) based one one-way ANOVA followed by Dunnett’s *post hoc* multiple comparison test. The differences between wildtype and mutant GFLV VSRs are marked by a line with corresponding asterisks. Different letters denote significantly different groups while the same letters denote treatments with *p* > 0.05. The error bars correspond to standard error means. These experiments were repeated three times with four to seven plants per treatment. **(J)** Western blot analysis of GFP accumulation using a specific anti-GFP monoclonal antibody. The bottom panel shows RuBisCO stained with the Pierce™ Reversible Protein Stain kit.

Plants treated with 1A^AG^, 1A^AG^B, and 1A^AG^B^GA^ consistently failed to suppress systemic *GFP* silencing in the apical leaves (0%, 0 /55), as shown by fluorescence imaging (Table 1; Figure 3A). A similar phenotype and suppression efficiency were observed in plants treated with the two RNA silencing controls (pHELL and pV; Table 1; Figure 3A). These results indicated that mutating W to A in the WG motif of 1A and 1AB abolished their ability to suppress systemic RNA silencing. Abolishment is defined here as a complete failure to inhibit RNA silencing in all biological replicates.

**Table 1:** Efficiency of systemic RNA silencing suppression by wildtype and mutant grapevine fanleaf virus (GFLV) suppressors of RNA silencing in co-infiltration assay with a *GFP* silencing inducing construct.

Treatment^a^	RNA silencing suppression efficiency^b^
Negative	0/10 = 0%
Mock	0/9 = 0%
pHELL	0/12 = 0%
pHELL + pV	0/16 = 0%
pHELL + GLRaV 2 p24	9/14 = 64%
pHELL + GFLV 1A	4/19 = 21%
pHELL + GFLV 1A^AG^	0/19 = 0%
pHELL + GFLV 1B	7/19 = 37%
pHELL + GFLV 1B^GA^	4/19 = 21%
pHELL + GFLV 1AB	5/22 = 23%
pHELL + GFLV 1A^AG^B	0/18 = 0%
pHELL + GFLV 1AB^GA^	3/18 = 17%
pHELL + GFLV 1A^AG^B^GA^	0/18 = 0%

a Negative: no treatment; Mock: infiltration buffer; pHELL: RNA silencing inducing construct pHELLSGATE8-EGFP; pV: empty vector control; and p24: positive control.

b Suppression efficiency was calculated across three independent experiments. Only plants that exhibited a complete supression of RNA silencing in the apical leaves were counted.

Systemic *GFP* silencing was observed in the apical leaves at 21% (4/19) and 17% (3/18) of the plants treated with 1B^GA^ or 1AB^GA^, respectively, as shown by fluorescence imaging (Table 1; Figure 3A). Plants that developed RNA silencing suppression upon the 1B^GA^ or 1AB^GA^ treatment are referred to as R-1B^GA^ or R-1AB^GA^ (R stands for reduced systemic RNA silencing suppression ability), respectively, in the remainder of the manuscript. In contrast, plants treated with 1A, 1B and 1AB and positive control p24 developed suppression of systemic *GFP* silencing with an efficiency greater than 20% (21%, 4/19 for 1A; 37%, 7/19 for 1B; 23%, 5/22 for 1AB; and 64%, 9/14 for p24; Table 1; Figure 3A). These results indicated that mutating W to A in the GW motif of 1B and 1AB reduced their ability of suppress systemic RNA silencing. Reduction is defined here as a limited ability to induce suppression of RNA silencing, resulting in a small proportion of biological replicates exhibiting the suppression phenotype. Interestingly, plants co-infiltrated with pHELL and 1B developed systemic silencing of *GFP* at 5–9 dpi, but the spread of systemic *GFP* silencing was abolished at 12–14 dpi, resulting in a complete restoration of *GFP* expression around 20 dpi (Supplementary Figure 2). The negative control plants (neg and mock) did not develop RNA silencing or suppression, as expected (Table 1; Figure 3A).

The failure to suppress RNA silencing by 1A^AG^, 1B^GA^, 1A^AG^B, 1AB^GA^, and 1A^AG^B^GA^ was validated by quantitative analyses of GFP fluorescence intensity and *GFP* expression via RT-qPCR (Figures 3B–I), as well as by semi-quantitative analysis of GFP accumulation (Figure 3J). The GFP fluorescence intensity and relative *GFP* expression levels in plants treated with mutant GFLV VSRs were not significantly different (*p* > 0.05) than those observed in RNA silencing control plants (pHELL and pV) but significantly lower than those in plants treated with wildtype GFLV VSRs (Figures 3B–I). In western blot analyses, GFP protein accumulated at lower levels in plants treated by mutant VSRs compared with wildtype VSRs (Figure 3J). The only exceptions were R-1B^GA^ plants, for which the GFP fluorescence intensity, *GFP* expression, and GFP accumulation levels were not significantly different (*p* > 0.05) or not lower than that of 1B-treated plants (*p* < 0.05; Figures 3C,G,J). Quantitative data obtained with R-1AB^GA^-treated plants were excluded for statistical analyses due to low sample numbers (*n* = 2; Supplementary Figure 3). As expected, GFP expression at the protein and transcript levels in wildtype GFLV VSRs-treated plants (1A, 1B, and 1AB) and p24-treated plants (Li et al., 2018; Choi et al., 2023) were significant higher in comparison with RNA silencing control-treated plants (pHELL and pV; Figures 3B–J). Together, these results showed that mutating (i) W of the WG motif in 1A and 1AB abolished their RNA silencing suppression activity and (ii) W of the GW motif in 1B and 1AB reduced their ability to suppress RNA silencing (Table 1; Figure 3).

### 3.3 Mutating the WG/GW motif of GFLV VSRs interferes with their ability to limit siRNA accumulation in the apical leaves

**Figure 4:**
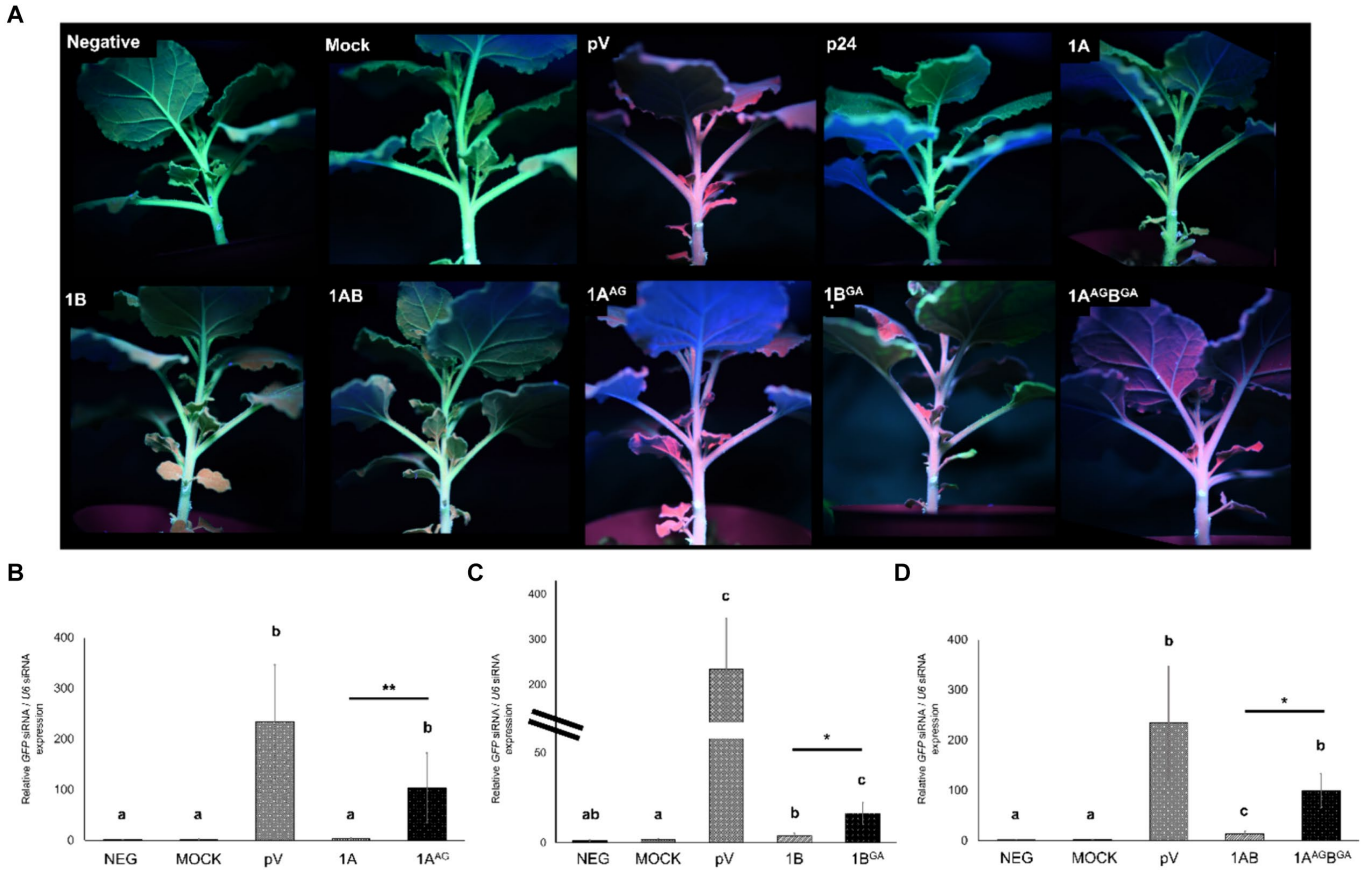
Effects of mutations in the WG/GW motifs of viral RNA silencing suppressors (VSRs) encoded by grapevine fanleaf virus (GFLV) on siRNA accumulation at the systemic level after 20 days post-infiltration. **(A)** Fluorescence images of *Nicotiana benthamiana* 16c line plants co-infiltrated with RNA silencing inducing construct pHELLSGATE8-EGFP (pHELL) and one of the wildtype (1A, 1B, and 1AB) or mutant (1A^AG^, 1B^GA^, and 1A^AG^B^GA^) GFLV VSRs. The empty vector (pV) and mock treatments were used as negative controls, and p24 of grapevine leafroll-associated virus 2 was used as a positive control. Photos were taken under a high-intensity UV lamp using a NIKON 850 digital camera. **(B–D)** Relative abundance of *GFP* siRNA normalized against the nuclear RNA *U6* siRNA and calibrated to untreated *N. benthamiana* 16c plants for plants treated with **(B)** 1A and 1A^AG^, **(C)** 1B and 1B^GA^, and **(D)** 1AB and 1A^AG^B^GA^. Three technical replicates were used per biological replicate (plant). The two lines for panel C represent split y-axis. The asterisks denote significant differences (*p* < 0.05*, *p* < 0.01**, and *p* < 0.001***) in siRNA accumulation compared with the average value of untreated *N. benthamiana* 16c and mock treatments according to Kruskal–Wallis test followed by pairwise Wilcoxon test. Difference between wildtype and mutant GFLV VSRs are marked by a line with asterisks. Different letters denote significantly different groups, while the same letters denote treatments with *p* > 0.05. The error bars correspond to standard error means. This experiment was repeated three times with two to four plants per treatment.

The effect of wildtype and mutant GFLV-GHu VSRs on siRNA accumulation was examined in the apical leaves of transgenic *N. benthamiana* 16c plants by fluorescence imaging, sRNA extraction with integrity assessment, and stem-loop RT-qPCR at 20 dpi (Supplementary Figure 4). Visual observation of the stems of mutant GFLV VSRs-treated plants showed the development of long-distance systemic spread of RNA silencing, while those treated with wildtype GFLV VSRs exhibited no systemic RNA silencing, as shown by fluorescence imaging (Figure 4A).

GFLV 1A^AG^-treated plants exhibited significantly higher (*p* < 0.01) systemic GFP siRNA accumulation with 2,769% difference in comparison with those detected in 1A-treated plants (Table 2; Figure 4B). Similarly, a significant (*p* < 0.05) increase in systemic GFP siRNA accumulation level was observed for 1B^GA^ and 1AB^GA^ in apical leaves with a relative increase in siRNA abundance of 305% and 619% in comparison with 1B and 1AB, respectively (Table 2; Figures 4C,D).

**Table 2:** Relative percentage increse in siRNA abundance between wildtype and mutant grapevine fanleaf virus (GFLV) RNA silencing suppressor-treated *Nicotiana benthamiana* 16c line plants.

RNA silencing^a^	Treatment^b^	Relative percentage increase in average siRNA abundance (%)^c^
−	Mock	14,379
+	pHELL + pV	
−	pHELL + GFLV 1A	2,769
+	pHELL + GFLV 1A^AG^	
−	pHELL + GFLV 1B	305
+	pHELL + GFLV 1B^GA^	
−	pHELL + GFLV 1AB	619
+	pHELL + GFLV 1A^AG^B^GA^	

a Establishment of RNA silencing (+) or no RNA silencing (−) in N. benthamiana 16c plants.

b RNA silencing inducing construct pHELLSGATE8-EGFP was co-infiltrated with control, wildtype or mutant GFLV VSRs. Mock was used as a negative RNA silencing control, and empty vector pV was used as a positive RNA silencing control.

c Percentage change between a wildtype and corresponding mutant treatment or between empty vector and mock.

Plants treated with 1A^AG^ and 1A^AG^B^GA^ exhibited relative GFP siRNA abundance values of 103.85 ± 68.78 and 99.38 ± 34.31, respectively (Figures 4B,C). Plants treated with 1B^GA^ had a relatively lower GFP siRNA abundance value (16.48 ± 5.89) in comparison with those treated with 1A^AG^ and 1A^AG^B^GA^, while those exhibiting the R-1B^GA^ phenotype had a similarly low value based on observation of numeric values without statistical analyses (19.96 ± 2.8; Figure 4; Supplementary Figure 5). As anticipated, negative controls (neg and mock) showed the lowest GFP siRNA abundance value (1.31 ± 0.52 and 1.62 ± 0.78, respectively), and pV-treated plants exhibited the highest relative GFP siRNA abundance value (234.56 ± 111.95; Figures 4B–D).

Together, these results showed that mutating the WG/GW motifs of GFLV VSRs interferes with their ability to reduce the accumulation of siRNA in apical leaves.

### 3.4 Mutating W in the WG/GW motifs changes the ability of GFLV VSRs to alter the expressions of host genes involved in RNA silencing

**Figure 5:**
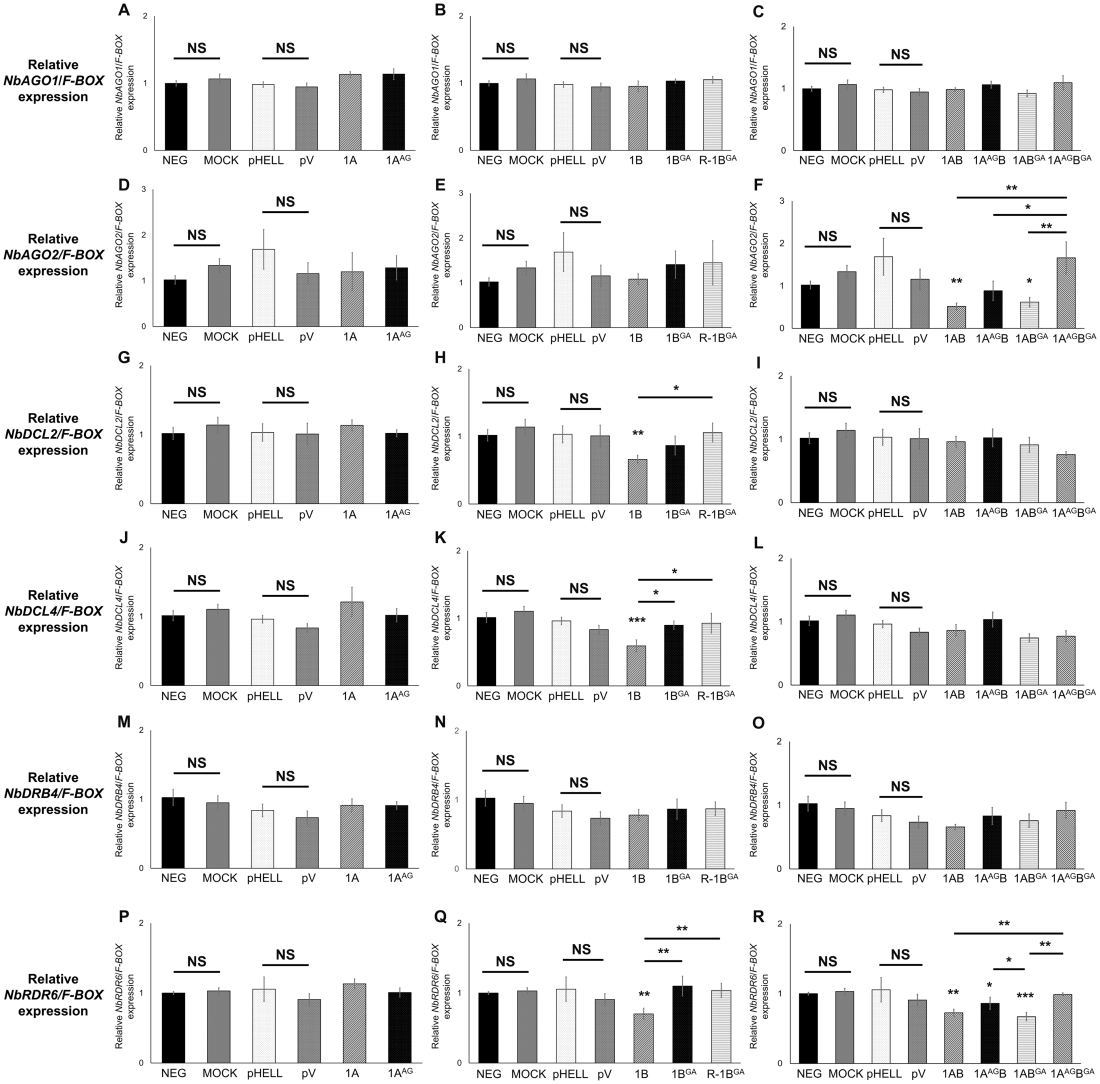
Effect of the WG/GW motif in RNA silencing suppressors (VSRs) of grapevine fanleaf virus (GFLV) on the expression of host genes involved in RNA silencing at the systemic level in *Nicotiana benthamiana* 16c line plants. Plants were co-infiltrated with RNA silencing-inducing construct pHELLSGATE8-EGFP (pHELL) and one of the wildtype (1A, 1B, and 1AB) or mutant (1A^AG^, 1B^GA^, 1A^AG^B, 1AB^GA^, and 1A^AG^B^GA^) GFLV VSRs. The empty vector (pV) and mock treatments were used as negative controls, and p24 of grapevine leafroll-associated virus 2 was used as a positive control. Leaf tissues were collected from the apical leaves after 20 days post-infiltration. **(A–R)** Relative gene expression level of **(A–C)** *NbAGO1*, **(D–F)** *NbAGO2*, **(G–I)** *NbDCL2*, **(J–L)** *NbDCL4*, **(M–O)** *NbDRB4*, and **(P–R)** *NbRDR6* normalized against the housekeeping gene *NbF-BOX* and calibrated to untreated *N. benthamiana* 16c plants. Three technical replicates were used per biological replicate (plant). The asterisks denote significant differences (*p* < 0.05*, *p* < 0.01**, and *p* < 0.001***) compared with the average value of untreated and mock-treated *N. benthamiana* 16c plants according to one-way ANOVA followed by Dunnett’s *post hoc* multiple comparison test with log transformation. The significance between two specific treatments is shown by asterisks. No significant difference between treatments is indicated by NS (*p* > 0.05). The error bars correspond to standard error means.

The effect of mutant GFLV-GHu VSRs on the expression of the selected host genes involved in RNA silencing. i.e., *NbAGO1*, *NbAGO2*, *NbDCL2*, *NbDCL4*, *NbDRB4*, and *NbRDR6*, was explored by RT-qPCR with specific primers (Supplementary Table 1). Wildtype and mutant GFLV VSRs did not affect the relative expression level of *NbAGO1* in comparison with the negative controls (neg and mock; Table 3; Figures 5A–C). Mutating WG alone or both WG and GW motifs of GFLV 1AB abolished their ability to downregulate the relative *NbAGO2* (2.3-fold) expression in comparison with the negative controls (Table 3; Figure 5F). In contrast, 1AB^GA^ downregulated *NbAGO2* by 1.9-fold (Table 3; Figure 5F). The GW mutation abolished the ability of GFLV 1B to downregulate the relative *NbDCL2* (1.6-fold), *NbDCL4* (1.7-fold), and *NbRDR6* (1.4-fold) expressions in comparison with controls (Table 3; Figures 5H,K,Q). Wildtype and mutant GFLV VSRs did not affect the relative expression level of *NbDRB4* in comparison with the negative controls (Table 3; Figures 5M–O). Mutating both WG and GW motifs of 1AB abolished its ability to downregulate the relative *NbRDR6* expression in comparison negative controls (Table 3; Figure 5R).

**Table 3:** Summary of host genes regulated by wildtype and mutant grapevine fanleaf virus (GFLV) suppressors of RNA silencing.

	Host genes^a^					
Treatment	*NbAGO1*	*NbAGO2*	*NbDCL2*	*NbDCL4*	*NbDRB4*	*NbRDR6*
GFLV 1A	-	-	-	-	-	-
GFLV 1A^AG^	-	-	-	-	-	-
GFLV 1B	-	-	Down 1.6	Down 1.7	-	Down 1.4
GFLV 1B^GA^	-	-	-	-	-	-
GFLV R-1B^GA^	-	-	-	-	-	-
GFLV 1AB	-	Down 2.3^b^	-	-	-	Down 1.4
GFLV 1A^AG^B	-	-	-	-	-	Down 1.2
GFLV 1AB^GA^	-	Down 1.9	-	-	-	Down 1.5
GFLV 1A^AG^B^GA^	-	-	-	-	-	-

a Significant downregulation in host gene expression relative to the average of the two negative controls (untreated and mock) is indicated as “down.” No significant differerence in expression relative to the average of two negative controls is indicated as “-”.

b The average fold change in comparison with the average of untreated and mock.

No significant alteration of host gene expression by 1A or 1A^AG^ was observed, while 1A^AG^B downregulated *NbRDR6* (1.2-fold), as did 1AB (1.4-fold; Table 3; Figure 5). GFLV 1AB^GA^ downregulated *NbRDR6* by 1.5-fold (Table 3; Figure 5R). As expected, no significant difference was observed between untreated and mock-treated plants, and between pHELL- and pV-treated plants (Figure 5). The positive control p24 significantly downregulated *NbAGO2*, *NbDCL2*, *NbDCL4*, and *NbRDR6* in comparison with negative and mock controls (Supplementary Figure 3).

These results showed that the GW motif in 1B and 1AB but not the WG motif in 1A and 1AB, with the exclusion of 1A^AG^B, is required to significantly alter the relative expression of *NbAGO2*, *NbDCL2*, *NbDCL4*, and *NbRDR6* (Table 3; Figure 5).

### 3.5 Residue W in positions 293 and 954 of polyprotein P1 affects GFLV infectivity *in planta*

**Figure 6:**
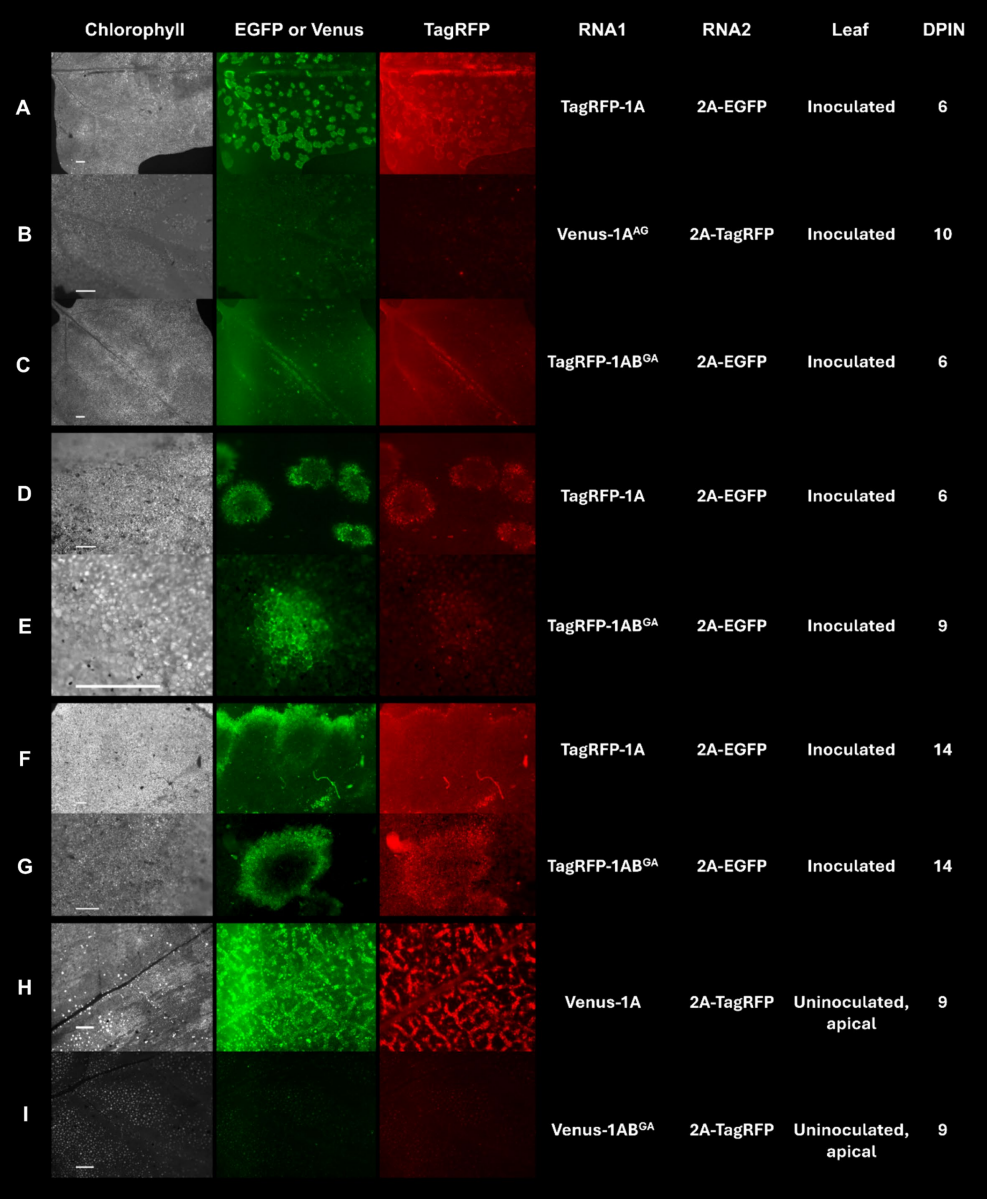
Infection of *Chenopodium quinoa* plants with grapevine fanleaf virus (GFLV) strain F13 (GFLV-F13) following inoculation with recombinant RNA1 and RNA2 transcripts tagged with specific fluorophores. **(A–I)** Expression of transcripts tagged with EGFP or Venus results in green fluorescence and those tagged with TagRFP results in red fluorescence. Leaves of *C. quinoa* were inoculated with **(A,D,F)** RNA1 transcript TagRFP-1A or **(C,E,G)** mutant RNA1 transcript TagRFP-1AB^GA^ (GW to GA mutation in protein 1B sequences) along with RNA2 transcript 2A-EGFP. **(B)** Mutant RNA1 transcript Venus-1A^AG^ (WG to AG mutation in protein 1A sequences) or **(H)** RNA1 transcript Venus-1A or **(I)** mutant RNA1 transcript Venus-1AB^GA^ was co-inoculated with RNA2 transcript 2A-TagRFP. Observations for virus infection were made in **(A–G)** inoculated leaves and **(H,I)** apical, uninoculated leaves with an Axiozoom V16 stereomicroscope (Zeiss). Scale bars are **(A–C,F,H,I)** 1 mm or **(D,E,G)** 500 μm. The first column panels shows the chlorophyll channel with an excitation of 625–655 nm and an emission of 665–715 nm. The second column panels show fluorescence photos taken with an excitation at 450–490 nm and an emission at 500–550 nm for EGFP and Venus. The third column panels show fluorescence photos taken with an excitation at 538–562 nm and an emission at 570–640 nm for TagRFP. The fourth and fifth columns show the corresponding recombinant RNA1 and RNA2 that were inoculated. The sixth and seventh columns show the type of leaf (inoculated or an uninoculated, apical leaf) that was visualized and the days post-inoculation (dpin), respectively.

Residue W in positions 293 and 954 of the polyprotein P1 encoded by the RNA1 of GFLV-F13 was mutated to A in recombinant transcripts derived from pMV13 (Viry et al., 1993) encoding either Venus (RNA1 Venus-1A) or TagRFP (RNA1 TagRFP-1A) to evaluate the effect of the substitution mutation on GFLV infectivity and movement *in planta*. Plants inoculated with GFLV-F13 transcripts RNA1 Venus-1A^AG^ (W to G mutation in 1A) and RNA2 2A-EGFP failed to develop infection in inoculated leaves (Figure 6B) or in apical, uninoculated leaves (data not shown). Plants inoculated with GFLV-F13 RNA1 TagRFP-1AB^GA^ (W to G mutation in 1B) and RNA2 2A-EGFP produced infection sites in inoculated leaves, although a delayed development was observed compared with plants inoculated with RNA1 TagRFP-1A and RNA2 2A-EGFP (Figures 6A,C,E,G), with no visible infection site at 6 days post-inoculation (dpin; Figure 6C), early infection at 9 dpin (Figure 6E) and full infection at 14 dpin (Figure 6G), as shown by a donut shape structure that is typical of GFLV infection (Schmitt-Keichinger et al., 2017). In apical, uninoculated leaves, no virus infection was present in plants inoculated with GFLV-F13 RNA1 Venus-1AB^GA^ and RNA2 2A-TagRFP via fluorescence observation in any of the inoculated plants, despite repeated experiments (Figure 6I). In contrast, infection with GFLV-F13 RNA1 and RNA2 transcripts without mutations resulted in infection sites readily visible in inoculated leaves at 3 dpin, and then showing a donut shape structure by 6 dpin (Figures 6A,D), and large coalescent sites at 14 dpin (Figure 6F), indicating a well-advanced infection stage. In uninoculated apical leaves, systemic infection by GFLV-F13 RNA1 Venus-1A and RNA2 2A-TagRFP transcripts was visible at 9 dpin (Figure 6H). These findings indicated that W in WG of 1A is essential for the infectivity of GFLV, while a W in GW of 1B is important for a timely development of infection in inoculated leaves and critical for systemic infection.

### 3.6 Predictive structural characteristics of WG and GW motifs in wildtype and mutant GFLV VSRs

Proteins 1A and 1B of GFLV strains GHu and F13 share 97 and 100% aa sequence identity, respectively (Vigne et al., 2013). The predicted structural location of WG in 1A overlapped for GFLV strains GHu and F13 in RoseTTAFold but not in AlphaFold2, while the location of GW in 1B overlapped for both GFLV strains in AlphaFold2 and RoseTTAFold (Supplementary Figure 6). For 1AB, the GW motif aligned for GLFV strains F13 and GHu in both AlphaFold2 and RoseTTAFold while WG did not (Supplementary Figure 6). TM-score values above 0.50 were obtained with AlphaFold2 between 1A and 1A^AG^ (0.59), 1B and 1B^GA^ (0.88), 1AB and 1A^AG^B (0.51), 1AB and 1AB^GA^ (0.59), and 1AB and 1A^AG^1B^GA^ (0.52), while TM-score values were below 0.50 with RoseTTAFold (Table 4). The IDTT confidence scores for AlphaFold2 and RoseTTAFold predictions were lowest for GFLV 1A and 1A^AG^ (Supplementary Figure 7).

**Table 4:** Template modeling (TM) score of predicted protein structures between wildtype and mutant grapevine fanleaf virus (GFLV) suppressors of RNA silencing via AlphaFold2 and RoseTTAFold.

	AlphaFold2^a^	RoseTTAFold^b^
GFLV wildtype and mutant VSRs	TM-score^c^	TM-score
1A	0.59	0.23
1A^AG^		
1B	0.88	0.45
1B^GA^		
1AB	0.51	0.39
1A^AG^B		
1AB	0.59	0.45
1AB^GA^		
1AB	0.52	0.43
1A^AG^B^GA^		

a Comparative similarity in protein structures predicted by AlphaFold2.

b Comparative similarity in protein structures predicted from RoseTTAFold.

c TM-score scale with 1 being a complete match between the two structures.

**Figure 7:**
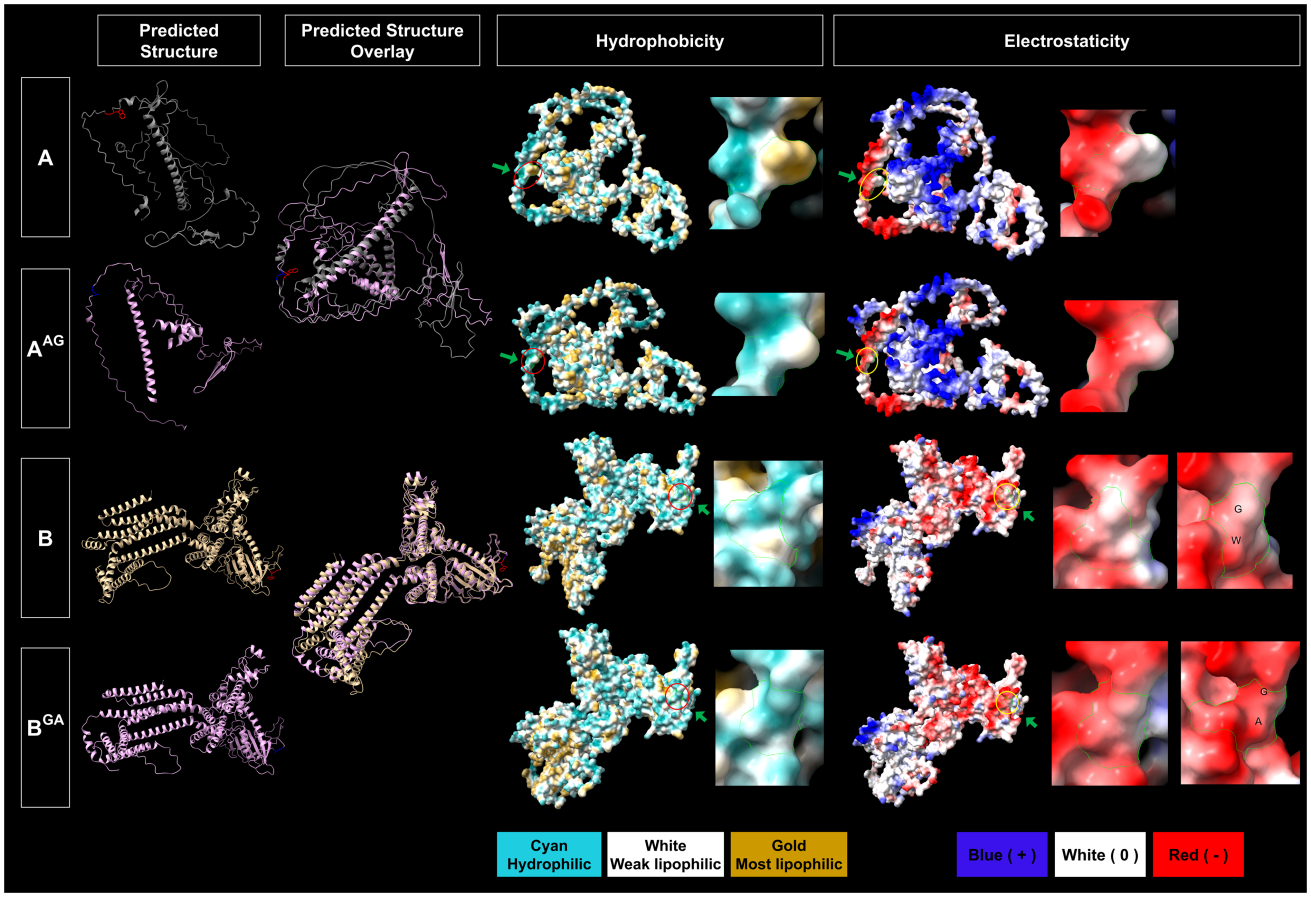
Predicted structural features of the WG and GW motifs of wildtype and mutant grapevine fanleaf virus (GFLV) proteins 1A and 1B. Protein structures were predicted via AlphaFold2 and analyzed via ChimeraX with the structural locations of WG and GW and their mutants shown in red (WG of 1A), blue (AG of 1A^AG^) for 1A, and in red (GW of 1B), and blue (GA of 1B^GA^) for 1B (first column). The overlay between predicted protein structures of wildtype 1A and 1B and their mutants are displayed (second column). The hydrophobicity of predicted protein structures is shown with cyan representing a hydrophilic surface, white representing a weak lipophilic surface, and gold representing a most lipophilic surface. The WG and GW motifs are marked with red circles and green arrows (third column). The electrostaticity of predicted protein structures is shown with blue representing a positively charged surface, white representing a surface with neutral charge, and red representing a negatively charged surface. The corresponding WG and GW motifs are marked with yellow circles and green arrows (fifth column). Close-up views of hydrophobicity (fourth column) and electrostaticity (sixth column) maps of WG, AG, GW, and GA are shown with green borderlines. Images were captured by ChimeraX.

**Figure 8:**
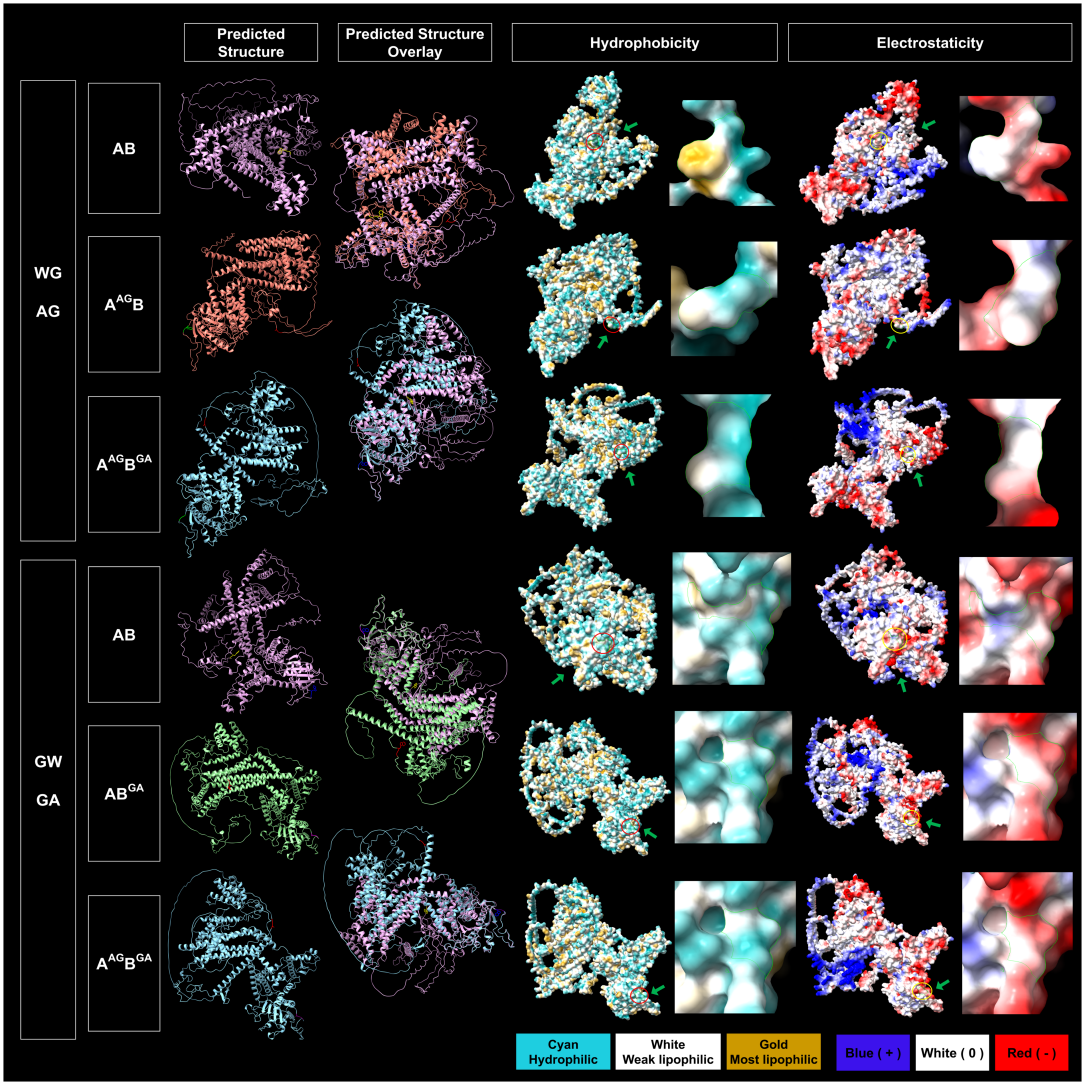
Predicted structural features of the WG and GW motifs of wildtype and mutated grapevine fanleaf virus (GFLV) protein 1AB. Protein structures were predicted via AlphaFold2 and analyzed via ChimeraX with the structural locations of WG and GW and their mutants shown in yellow (WG of 1AB), red (AG of 1A^AG^B and 1A^AG^B^GA^), blue (GW of 1AB), and purple (GA of 1AB^GA^ and 1A^AG^B^GA^; first column). The overlay between predicted protein structures of GFLV wildtype 1AB and its mutants are displayed (second column). The hydrophobicity of predicted protein structures is shown with cyan representing a hydrophilic surface, white representing a weak lipophilic surface, and gold representing a most lipophilic surface. The WG and GW motifs are marked with red circles and green arrows (third column). The electrostaticity of predicted protein structures is shown with blue representing a positively charged surface, white representing a surface with neutral charge, and red representing a negatively charged surface. The corresponding WG and GW motifs are marked with yellow circles and green arrows (fifth column). Close-up views of hydrophobicity (fourth column) and electrostaticity (sixth column) maps of WG, AG, GW, and GA motifs are presented in the right panels marked with green borderlines for each section. Images were captured by ChimeraX.

*In silico* protein structure prediction by AlphalFold2 further revealed that WG of 1A and GW of 1B are exposed on the surface of the proteins with closed (facing inward) and open (facing outward) conformations, respectively, for GFLV-GHu (Figure 7). For 1AB, the WG motif is located at the protein interior with closed conformation, while the GW motif is surface exposed with open conformation (Figure 8). The surface exposed WG/GW motif could favor site-specific interaction with host factors, while closed WG/GW conformations could favor intramolecular interactions for protein stability (Robertson, 2002; Faccio, 2018). A WG motif localization on the protein exterior with open conformation was also observed for p24 of GLRaV2 (Supplementary Figure 8).

*In silico* substitution of W with A in the WG motif changed the surrounding residues of the WG from both negative and positive charged and hydrophobic residues to less positive and hydrophobic residues for 1A^AG^, possibly interfering with hydrophilic-mediated interactions and aromatic residue-dependent bindings (Yan et al., 2003; Lingel et al., 2004; Pfaff and Meister, 2013; Supplementary Figure 9). Furthermore, the WG motif of GFLV 1AB is closely surrounded by a positive and neutral charged and hydrophobic surface; this is not the case for the AG of 1A^AG^B (Supplementary Figure 9). The WG to AG mutation changes the predicted structural localization of WG motif from protein interior to surface and from closed to open conformation for 1A^AG^B but not 1A^AG^, potentially interfering with protein stability (Figures 7, 8; Gromiha and Selvaraj, 2004). However, this mutation reduced hydrophobicity but did not alter electrostaticity within the WG motif in 1A^AG^ and 1A^AG^B. This result was expected as A is less hydrophobic than W, and W and A are both neutrally charged (Figures 7, 8).

*In silico* substitution of GW to GA did not alter its surface exposed localization nor the open conformation but reduced hydrophobicity in 1B^GA^ and 1AB^GA^ (Figures 7, 8). Unexpectedly, although both A and W have neutral charges, this mutation potentially altered the motif surface charge from neutral to negative in 1B^GA^, possibly alternating hydrophilic interactions at the protein surface (Figure 7). There was no change in the surrounding negative and neutral charged and hydrophilic residues of the GW motif in 1B and 1AB when mutated from GW to GA (Supplementary Figure 9).

The double *in silico* mutation in WG and GW of 1AB changed the structural localization AG motif of 1A^AG^B^GA^ towards the surface with open conformation but not the GA motif (Figure 8). This mutation reduced the hydrophobicity but did not alter the electrostaticity of WG/GW motifs in 1A^AG^B^GA^ (Figure 8). In contrast, the AG of 1A^AG^B^GA^ was surrounded by negatively charged and hydrophobic residues, and, like the GW of 1AB, the GA of 1A^AG^B^GA^ was surrounded by negative and neutral charged and hydrophilic residues (Supplementary Figure 9).

The *in silico* protein structure prediction work via AlphaFold2 analyses showed that mutating W within WG/GW of the three GFLV VSRs predictably alters the protein structure, the location and conformation of the WG motif within the protein structure, and the physical and chemical properties of the proteins. Such changes might affect the functionality of the GFLV VSRs.

## 4 Discussion

In this study, we explored the role of the conserved WG/GW motif in the RNA silencing suppression function of the three GFLV VSRs: proteins 1A, 1B, and 1AB (Choi et al., 2023). We showed that mutating W of the WG/GW motif (i) either abolishes or reduces their ability to induce systemic RNA silencing suppression, (ii) eliminates their ability to limit siRNA accumulation, (iii) hinders their ability to downregulate *NbAGO2*, *NbDCL2*, *NbDCL4*, and *NbRDR6*, (iv) results in a noninfectious GFLV or a virus deficient in systemic movement *in planta*, and (v) changes the predicted chemical and physical properties of the VSR proteins.

The conserved WG/GW motifs were identified in GFLV proteins 1A (WG at aa positions 293–294), 1B (GW at aa positions 537–538), and 1AB (WG and GW motifs at aa positions 293–294 and 953–954, respectively) across GFLV strains (Figure 1). These motifs were also found at various locations in polyprotein P1 of several other nepoviruses, however, only a few of them encoded WG/GW motifs at the same aa positions as GFLV VSRs (Supplementary Table 3; Supplementary Figure 1). These results revealed that the presence of the conserved WG/GW motifs in GFLV VSRs are virus-specific but not genus-specific. Such relationship between the WG/GW motifs and VSR functions at a virus-specific level has been observed for nepoviruses. The CP of ToRSV is a VSR that requires the WG/GW motif for its suppression function through destabilizing AGO1 (Karran and Sanfaçon, 2014), while the CP of GFLV lacks a WG/GW motif and suppression ability (Choi et al., 2023). This demonstrates that the presence of a conserved silencing suppression-associated WG/GW motif is virus-dependent. In addition, the nepovirus proteins with WG/GW motifs may or may not endure silencing suppression abilities, as virus proteins with multiple WG/GW motifs lacking silencing suppression abilities have been reported (Giner et al., 2010; Szabó et al., 2012).

Predicted protein structure comparison between 1A and 1A^AG^ showed a similar protein fold (TM score > 0.5) for AlphaFold2 (TM score of 0.59) but not for RoseTTAFold (TM score of 0.23; Table 4). However, the W to A mutation in the WG motif of 1A predictably reduced its surface hydrophobicity and dramatically decreased the presence of surrounding positive and hydrophobic residues (Figure 7; Supplementary Figure 9). The hydrophobicity reduction was expected as A is less hydrophobic than W (Nozaki and Tanford, 1971; Luan et al., 1992; Chernov and Komatsu, 1995). Predicted changes in the physical and chemical properties due to a mutation in the WG motif of 1A may alter interactions with host factors, such as GW-containing proteins or sRNA, leading to 1A^AG^ lacking an RNA silencing suppression ability (0%, 0/19 plant) and siRNA-limiting capacity with an increase in siRNA abundance of 2,769% (Tables 1, 2; Figures 3, 4, 7; Supplementary Figure 9). In support of this hypothesis, the PAZ and MID domains of AGO protein bind to the 3′ and 5′ ends of sRNA, respectively, and the positively charged RNA central cleft structure of a specific loop of AGO is important for sRNA binding (Lingel et al., 2004; Ma et al., 2005; Parker et al., 2005; Poulsen et al., 2013; Jin et al., 2022). In addition, the interaction in the PAZ domain of AGO2 with protein and nucleic acid is formed through aromatic residues, like residue W, in the hydrophobic cleft structure for *Drosophila melanogaster* (Lingel et al., 2003; Yan et al., 2003; Lingel et al., 2004). Furthermore, the W-binding pocket of human AGO is responsible for interaction with GW182 protein, which is required for miRNA-mediated silencing (Meister et al., 2005; Behm-Ansmant et al., 2006; Pfaff et al., 2013; Jonas and Izaurralde, 2015). Changes in the charge and aromatic residue in 1A could explain functional differences between the wildtype and mutant VSR. Finally, the expression of six host genes (*NbAGO1, NbAGO2, NbDCL2, NbDCL4, NbDRB4, and NbRDR6*) involved in RNA silencing was not altered by 1A or 1A^AG^ (Table 3; Figure 5). It would be interesting to test if 1A alters *DRB2* expression because it is upregulated upon GFLV-GHu infection (Roy et al., 2023) though increased *DRB2* expression was shown to decrease GFLV accumulation (Incarbone et al., 2021). It is possible that 1A directly interacts with host factor(s) for RNA silencing suppression instead of manipulating host gene expression.

*In silico* protein structure prediction and comparative analyses between 1B and 1B^GA^ resulted in the least protein structure variation (TM score of 0.88 for AlphaFold2 and 0.45 for RoseTTAFold) in comparison with other wildtype and mutant GFLV VSRs comparisons (Table 4). Mutating the GW motif of 1B did not alter its surface-exposed and open conformation structure but, as anticipated, lowered its hydrophobicity (Figure 7; Supplementary Figure 9). Because the net charge of both W and A is neutral, the change in predicted surface charge of 1B^GA^ from neutral to negative was unexpected (Figure 7). However, it is important to note that the GW motif of 1B is surrounded by negatively charged hydrophilic residues (Supplementary Figure 9). The charged and hydrophilic surrounding was proposed to be critical for exposing the W residue of the WG/GW motif on the protein surface for binding accessibility (Karlowski et al., 2010; Pfaff and Meister, 2013; Khemaissa et al., 2021). In addition, one of the most common catalytic residues for various enzymes (e.g., hydrolase, ligase, etc.) is W, which mainly functions as an electrostatic stabilizer or destabilizer that is important in controlling enzyme catalysis (Holliday et al., 2009). In addition, the stabilizer residues play an important role in facilitating interactions, mostly electrostatic reactions, with other residues for proper protein folding and thermal stability (Khemaissa et al., 2021). It is possible that W to A mutation of GW disrupts catalysis action or protein stability of 1B. Despite the negative charge of GA in 1B^GA^, the ability to induce RNA silencing suppression was maintained at low efficiency (Table 1; Figures 3, 7). The interaction between 1B and host factors for RNA silencing suppression might be facilitated by both hydrophilic interaction and W-binding. This might explain why 1B has a higher ability (37%, 7/19 plants) to suppress systemic RNA silencing compared with other GFLV VSRs (Choi et al., 2023). It is possible that, with W being substituted with A, GFLV 1B^GA^ binding with host factors is less stable, although weakly occurring via a hydrophilic surface, thus resulting in a reduction but not abolishment of RNA silencing suppression. It is important to note that, although prediction modeling provided insights into structural features of the WG/GW motif in GFLV VSR, the overall low confidence scores (Table 4; Supplementary Figure 7) stress the need for experimental validation.

Among all wildtype and mutant GFLV VSRs, protein 1B^GA^ exhibited the lowest percent change (305%) in *GFP* siRNA abundance in comparison with wildtype VSR (Table 2; Figure 4). This result may indicate that 1B^GA^ has a weak capacity at limiting siRNA accumulation, providing context to its ability to suppress RNA silencing at a low efficiency (21%, 4/19 plant; Tables 1, 2; Figures 3, 4). As the amplification of RNA silencing depends on the downstream RNA silencing pathway, specifically on secondary siRNAs derived from dsRNAs produced by RDR6-SGS3 (Gaffar and Koch, 2019; Jin et al., 2022; Lopez-Gomollon and Baulcombe, 2022), it is possible that 1B interferes with the amplification or movement of secondary siRNAs because the systemic movement of siRNA decreased over time (Supplementary Figure 2). As GFLV replication occurs on endoplasmic reticulum (ER)-derived vesicles (Ritzenthaler et al., 2002), it is possible for GFLV 1B to localize to the ER through its transmembrane domains and eventually sequester miRNA/siRNA through its predicted nucleotide-binding sites (S367, Q368, S369, K371, T372, and I373; Roy et al., 2024). This hypothesis is supported by the presence of dsRNA intermediates of replication in the ER-derived GFLV compartments in perinuclear space (Ritzenthaler et al., 2002). Moreover, miRNA-mediated translation repression has been documented to locate in the ER where AGO1 is localized in *Arabidopsis thaliana* leaves (Li et al., 2013). As W is important for stability and folding of membrane proteins, the GW mutation may interfere with the potential subcellular localization in the ER of 1B. More work is needed to assess whether predicted nucleotide-binding sites and transmembrane domains are critical for the suppression function of GFLV 1B.

In contrast to GFLV 1B, 1B^AG^-treated plants, as well as those of R-1B^AG^, did not exhibit downregulated expression of *NbDCL2*, *NbDCL4*, and *NbRDR6* in comparison with negative controls (Table 3; Figures 5H,K,Q), This result suggested no association between WG/GW motif-dependent RNA silencing suppression and alteration of *NbDCL2*, *NbDCL4*, and *NbRDR6* expression. However, this does not exclude the possibility of an effect at the protein level. Although the W in the WG motif of 1B is required to downregulate *NbDCL2*, *NbDCL4*, and *NbRDR6,* other WG/GW motif-associated host factors should be explored to further understand how 1B interferes with RNA silencing. One hypothesis is that 1B downregulates *NbDCL2*, *NbDCL4*, and *NbRDR6* expression to decrease secondary siRNA production through the miRNA pathway. Indeed, P38 of turnip crinkle virus (TCV, genus *Betacarmovirus*, family *Tombusviridae*) reduces *DCL4* expression by downregulating DCL1-targeting miR162 (Azevedo et al., 2010). In *Arabidopsis*, the accumulation of vsiRNAs decreases at both the local and systemic levels in the absence of *DCL2* and *DCL4* (Garcia-Ruiz et al., 2010). It would be interesting to explore how downregulating *NbDCL2* and *NbDCL4* expression affects systemic RNA silencing suppression efficiency of GFLV VSRs because *NbDCL2* downregulation reduces robustness of systemic RNA silencing, yet *DCL4* downregulation elevates it in *N. benthamiana* (Chen et al., 2018).

GFLV 1AB^GA^ had the least predicted structure variation (TM score of 0.59 for AlphaFold2 and 0.45 for RoseTTAFold) in comparison with 1AB, while 1A^AG^B (TM score of 0.51 for AlphaFold2 and 0.39 for RoseTTAFold) and 1A^AG^B^GA^ (TM score of 0.52 for AlphaFold2 and 0.43 for RoseTTAFold) had the most predicted structure variation (Table 4). Mutating W in the WG motif of 1AB eliminated its surface-exposed structural feature but not its closed conformation and decreased surrounding positive and hydrophobic surface properties (Figure 8; Supplementary Figure 9). Single or double mutations of the WG/GW motifs did not alter the electrostaticity of GFLV 1AB but led to a reduced surface hydrophobicity (Figure 8). A decrease in the positive charge and hydrophobicity may interfere with the binding to host factors, such as sRNA, causing an abolishment of the RNA silencing suppression ability of 1A^AG^B (0%, 0/18 plants) and 1A^AG^B^GA^ (0%, 0/18 plants; Table 1; Figure 3). Indeed, the dsRNA-binding domain 2 (dsRBD2) of human TAR RNA-binding protein (TRBP), a partner protein for DCL, contains a structural loop that is critical for the formation of a hydrophobic pocket with its nearby residues through hydrophobic and cationic interactions and binds to siRNA (Yamashita et al., 2011). In addition, *Arabidopsis* DCL3 binds to the 5′ of siRNA through its positively charged pocket surface within the platform-PAZ-connector domains, while the 3′ end of sRNA is bound by cap composed of aromatic amino acids in its PAZ domain (Wang et al., 2021). Mutations in such sRNA binding sites decrease siRNA production (Wang et al., 2021). Similar changes in physical and chemical properties of 1AB could influence its VSR function.

The relative negative net charge of 1AB^GA^ mildly increased but the hydrophobicity of its surrounding properties was unchanged in comparison with 1AB (Supplementary Figure 9). The GW motif of 1AB is surrounded by hydrophilic residues, as observed for the GW motif of 1B. This may favor W accessibility on the surface of the protein (Figure 8; Supplementary Figure 9; Karlowski et al., 2010; Pfaff and Meister, 2013). As similarly proposed for 1B, the GW of 1AB may target host factors through W-binding and hydrophilic interaction to suppress RNA silencing (23%, 5/22 plants; Table 1; Figure 3). The abolishment of the RNA silencing suppression ability of 1A^AG^B^GA^ (0%, 0/18 plants) might be due to the WG mutation outperforming the GW mutation (Table 1; Figure 3), and the 619% difference in *GFP* siRNA accumulation between 1AB and 1A^AG^B^GA^ could be explained by insufficient hydrophobic and cationic interactions with host factors, as discussed above (Table 2; Figures 4A,D, 8; Supplementary Figure 9). The systemic siRNA accumulation and production depend on efficiencies of secondary siRNA production, long-distance movement, and perception by receiving cells (Gaffar and Koch, 2019; Jin et al., 2022; Lopez-Gomollon and Baulcombe, 2022; Yan and Ham, 2022). It is unclear how mutations in WG and GW of 1AB lead to increase in systemic siRNA accumulation. Exploring the role of 1AB in secondary siRNA production, accumulation, and movement (Gaffar and Koch, 2019; Jin et al., 2022; Lopez-Gomollon and Baulcombe, 2022; Yan and Ham, 2022) would be interesting to shed light on how this VSR counters the plant innate immune system. More work is needed to address these issues.

GFLV 1A^AG^B and 1A^AG^B^GA^ lost their ability to downregulate *NbAGO2* expression, and 1A^AG^B^GA^ was defective in reducing the *NbRDR6* expression compared with 1AB (Table 3; Figures 5F,R). In contrast, the ability to decrease the relative *NbAGO2 and NbRDR6* expression was not altered for 1AB^GA^ (Table 3; Figures 5F,R). Together, these results suggested that the WG motif of 1AB is required to reduce *NbAGO2* expression, while either WG or GW is necessary to downregulate *NbRDR6* expression (Table 3; Figure 5). The AGO protein family targets and cleaves or represses translation of complementary RNA (Brodersen and Voinnet, 2006; Lopez-Gomollon and Baulcombe, 2022). The plant AGO2 is known for its antiviral function, especially against RNA viruses like TCV, tobacco rattle virus (genus *Tobravirus*, family *Virgaviridae*), turnip mosaic virus (genus *Potyvirus*, family *Alphaflexiridae*), and SPMMV (Harvey et al., 2011; Zhang et al., 2012; Carbonell and Carrington, 2015; Garcia-Ruiz et al., 2015). AGO2 functions hierarchically and antagonistically with AGO1 (Carbonell and Carrington, 2015). For example, AGO1 reduces AGO2 expression through the miR403 pathway (Carbonell and Carrington, 2015). It would be interesting to identify and measure the expression of miRNAs upon plant treatment with wildtype and mutant GFLV VSRs, as an induction of specific miRNA by p19 of Cymbidium ringspot virus (genus *Tombusvirus*; family *Tombusviridae*) has been reported (Várallyay et al., 2010). Knowing that RDR6 propels hairpin-induced RNA silencing during the RNA replication process (Harmoko et al., 2013), a reduction of *RDR6* expression may result in a high frequency of RNA silencing suppression by 1B and 1AB. These two GFLV VSRs may function similarly to Pns10 of rice dwarf phytoreovirus (genus *Phytoreovirus*, family *Reoviridae*) that reduces RDR6 expression and prevents the perception of siRNA into receiving tissues (Ren et al., 2010).

A downregulation of some host genes involved in plant RNA silencing by 1B and 1AB (Table 3; Figure 5) is inconsistent with a previous report on GFLV VSRs (Choi et al., 2023). Differences in experimental conditions could account for these inconsistencies with infiltration of GFLV VSRs being performed in the previous study after systemic RNA silencing was established (Choi et al., 2023). In this study, GFLV VSRs were introduced concurrently with the RNA silencing inducer pHELL. Differences in the temporal delivery of GFLV VSRs in relation to the onset of RNA silencing are likely changing the host responses, VSR behavior, and complex interactions among host factors and VSRs. Nonetheless, mutating WG/GW in GFLV VSRs resulted in an abolishment or a reduction in the ability to induce RNA silencing suppression. Similar results were reported for other plant VSRs, including the CP of ToRSV (Karran and Sanfaçon, 2014), P1 of SPMMV (Giner et al., 2010), p24 of GLRaV 2 (Li et al., 2018), p37 of PLPV (Pérez-Cañamás and Hernández, 2015), and p38 of TCV (Azevedo et al., 2010). However, not all WG/GW motifs are required for VSR suppression activity. For example, mutating one of three GW motifs to GA in Pro2Glu of strawberry mottle virus (SMoV, genus *Stramovirus*, family *Secoviridae*) did not result in a loss or a reduction of suppression activity (Fan et al., 2022). Similarly, mutating GW to GA in p28 of SMoV did not abolish its suppression activity (Fan et al., 2022). Moreover, the artificial introduction of WG/GW motifs via substitution mutagenesis can result in a gain of RNA silencing suppression activity for some VSRs. For example, protein P1 of sweet potato feathery mottle virus (genus *Potyvirus*, family *Potyviridae*) does not have a recognized silencing suppression activity but a double mutation of GH and GY to GW in protein P1 results in a gain of local suppression activity (Szabó et al., 2012).

Several VSRs interfere with interactions between AGOs and GW/WG motifs-containing host proteins involved in RNA silencing like silencing defective 3, SPT5-like elongation factor, and RNA polymerase V subunit NRPE1 (El-Shami et al., 2007; Bies-Etheve et al., 2009; Garcia et al., 2012; Xu et al., 2020). For example, the 2b of cucumber mosaic virus (genus *Cucumovirus*, family *Bromoviridae*) and P38 of TCV directly bind to AGO1 and inhibit its functions (Zhang et al., 2006; Azevedo et al., 2010). The P25 of potato virus X (genus *Potexvirus*, family *Alphaflexiviridae*) and CP of ToRSV bind and induce degradation of AGO1 (Chiu et al., 2010; Karran and Sanfaçon, 2014). It would be interesting to examine if the WG/GW motifs of GFLV VSRs bind with AGO1 and/or siRNA, or other host proteins that interact with AGOs (El-Shami et al., 2007; Bies-Etheve et al., 2009; Garcia et al., 2012; Pérez-Cañamás and Hernández, 2015; Gupta and Tatineni, 2019; Xu et al., 2020; Fan et al., 2022).

Mutations in the GW/WG motifs in some VSRs limit or inhibit the establishment or progression of virus infection, as observed in P38 of TCV (Azevedo et al., 2010) and p37 of PLPV (Pérez-Cañamás and Hernández, 2015). Virus infectivity experiments with GFLV-F13 *in planta* showed that the W to A mutation in WG of 1A resulted in no detectable infection in either inoculated or apical leaves, indicating that the virus was impaired at the cellular level (Figure 6B). This result was unexpected because no RNA silencing suppression activity was previously detected at the local level (Choi et al., 2023). This obviously does not conclude that 1A has no ability to suppress RNA silencing at the local level, and the influence of different experimental conditions on this potential effect warrants further exploration. A defect in the establishment of infection by GFLV-F13 encoding 1A^AG^ might be due to a loss of its RNA silencing suppression ability or an unidentified role in host invasion (Figure 6B). More work would also be needed to determine whether GFLV-F13 with 1A^AG^ is capable of replicating in protoplasts. Replacing W with A in the GW motif of 1B delayed GFLV-F13 infection at the local level and prevented systemic infection (Figures 6C,E,G,I), suggesting that 1B might be involved in host invasion, like many other VSRs, by acting at the level of initially infected cells and their neighboring cells, as well as in distant tissues (Liu et al., 2023). The ability of GFLV 1B^GA^ to suppress systemic RNA silencing was reduced (Table 1; Figure 3), potentially explaining why mutating the GW motif in 1B delays rather than inhibits the establishment of virus infection *in planta* (Figure 6), whereas mutating the WG motif in 1A results in a complete loss of suppression ability and virus infectivity (Figures 3, 6). Work with GFLV encoding 1A^AG^ and/or 1B^GA^ was performed with strain F13. Similar results are anticipated with GFLV-GHu because both strains have been previously shown to suppress RNA silencing (Choi et al., 2023).

In summary, our results show that the WG/GW motif encoded by GFLV VSRs is required to endure the ability to suppress systemic RNA silencing and establish virus infection. These findings open new avenues for the functional characterization of GFLV VSRs and the unraveling of how GFLV counteracts the host innate immunity.

## Data availability statement

The original contributions presented in the study are included in the article/Supplementary material, further inquiries can be directed to the corresponding authors.

## Author contributions

JC: Conceptualization, Data curation, Formal analysis, Investigation, Methodology, Project administration, Supervision, Validation, Visualization, Writing – original draft, Writing – review & editing. SB: Investigation, Writing – review & editing. CS-K: Conceptualization, Methodology, Project administration, Resources, Supervision, Writing – review & editing, Data curation, Formal analysis, Investigation, Validation, Visualization. MF: Conceptualization, Methodology, Project administration, Resources, Supervision, Validation, Writing – review & editing, Funding acquisition, Writing – original draft.
