# Supplementary material for: Mutations in the WG and GW motifs of the three RNA silencing suppressors of grapevine fanleaf virus alter their systemic suppression ability and affect virus infectivity
Source: Front Microbiol. 2024 Aug 12;15:1451285. doi: 10.3389/fmicb.2024.1451285 (PMC11345138; doi:10.3389/fmicb.2024.1451285)
Supplement: Supplementary file 1 [file Data_Sheet_1.PDF]

Supplementary Table 1. Primers used in this study for site-directed mutagenesis PCR, RT-qPCR, and siRNA quantification.

Experiment[a]	Primer name	Primer Sequence (5′ - 3′)[b]	Reference
Site-directed mutagenesis PCR	GFLV GHu 1A-W293A-FOR	GGACTTTGATgctGGGGAGAAGTC	This study
	GFLV GHu 1A-W293A-REV	TCTAAGCAGGAAAAGTCC	
	GFLV GHu 1B-W538A-FOR	GGATGAGGGAgctATCACTCCGAG	
	GFLV GHu 1B-W538A-REV	ATAGGAGTATTTACTGCAG	
GFLV strain F13 RNA1 subcloning and mutagenesis PCR	353LR1ST7	TCAGAGTCGACTAATACGACTCACTATAGATGAAAATTTCCCACAAGTTCTTACG	This study
	29AvrNt1AR	GGCACCTGCCACATCCTAGGAGTGGCAAACAA	
	28AvrNtF	TTGTTTGCCACTCCTAGGATGTGGCAGGTGCC	
	302LRRI798	GGACGACTTTCCCCTTAGTG	
	297AvrVenusF	GGGGCCTAGGATGGTGAGCAAGGGCGAGGA	
	298AvrVenusR	GGGGCCTAGGCTTGTACAGCTCGTCCATGCCG	
	132AvrTagRFPF	GGGGTACCCCTAGGATGAGCGAGCTGATTAAGGAG	
	133AvrTagRFPR	GGGGTACCCCTAGGATTAAGTTTGTGCCCCAGTTTGC	
	4981BW954A	GCGATCACTCCAAGTGAGGCTGTTGCAG	
	4991Bseq3100R	TCCTTCATCCATGGGAGTGTTTAC	
RT-qPCR	*NbAGO1*-FOR	TTCGTCCTACACAGATGGGA	Tarquini et al., 2021
	NbAGO1-REV	AGAAGCTGGCTCACAAAGTC	
	NbAGO2-FOR	CATTTGAACCTCCTTTCTATCGAC	Liu et al., 2012
	NbAGO2-REV	CATACCTCTAGAAGTGAGGATCAC	
	NbDCL2-FOR	GAAGAACCACTTCTTAGGGGAAA	Dadami et al., 2013
	NbDCL2-REV	GGCCATAACAAGGACTCAA	
	NbDCL4-FOR	ACTTCTACGCAGTAGGCCGT	Choi et al., 2023
	NbDCL4-REV	CGCAACCACTGATGACATTTGC	
	NbDRB4-FOR	CCCGAAAAACAGACCTTGCC	This Study[c] Reference Sequence Niben101Scf05841
	NbDRB4-REV	GTCACAGAATTTGCTTCTGCCA	
	NbF-BOX-FOR	GGCACTCACAAACGTCTATTTC	Liu et al., 2012
	NbF-BOX-REV	ACCTGGGAGGCATCCTGCTTAT	
	GFP-FOR	AGCAGAAGAACGGCATCAAG	Choi et al., 2023
	GFP-REV	GTGCTCAGGTAGTGGTTGTC	
	NbRDR6-FOR	AGTGATCTAGCAACCCAATGAG	Liu et al., 2012
	NbRDR6-REV	TTCAGGAATGTCTTCGAGCG	
siRNA quantification	GFP-stem-loop	GTCGTATCCAGTGCAGGGTCCGAGGTATTCGCACTGGATACGACGCCGTT	Tarquini et al., 2021
	U6-stem-loop	GTGCAGGGTCCGAGGTTTTGGACCATTTCTCGAT	Turner et al., 2013
	GFP-siRNA-qPCR-FOR	TTCCTGCCGACAAGCAAAAG	Tarquini et al., 2021
	U6-siRNA-qPCR-FOR	GGAACGATACAGAGAAGATTAGCA	Turner et al., 2013
	Universal-qPCR-REV	GTGCAGGGTCCGAGGT	Varkonyi-Gasic et al., 2007

[a]Primers were divided into four different categories based on the experiment performed.
[b]For site-directed mutagenesis, the nucleotide sequences that have been mutated via substitution are shown in lowercase. GFLV stands for grapevine fanleaf virus.
[b]Reference sequence from the *Nicotiana bethamiana* v1.0.1. database used to design *NbDRB4* in this study.

Supplementary Table 2. Sequences of grapevine fanleaf virus (GFLV) RNA1 used in this study.

GFLV isolate	GenBank accessions
F13	NP_619689
GHu	AMF91094
WAPN173	ACZ58632
WAPN6132	ACZ58633
SAPCS3	AFI71813
SACH44	AGT42200
WTR4	ARO69845.1
WTR6	ARO69851
SWT2 clone 1	ARO69856
SWT2 clone 3	ARO69858

Supplementary Table 3. Location of tryptophan-glycine (WG) and glycine-tryptophan (GW) motifs in nepovirus RNA1-encoded polyprotein.

GenBank Reference[a]	Name[b]	WG[c]	GW[d]
AAQ73822.1	RrRSV	659-660	2-3, 130-131, 465-466, 715-716, 1495-1496
APO15117.1	SLSV	1164-1165, 1214-1215	735-736, 769-770, 1480-1481
BAL04700.1	BLSV	85-86	700-701, 734-735, 1749-1750
AQT41504.1	PRMV	85-86, 427-428, 1963-1964	696-697, 730-731
CCA64538.1	CLRV	197-198, 1322-1323	198-199, 581-582, 2059-2060
AAA78254.1	ToRSV	1075-1076	2060-2061, 2138-2139
BAB89369.1	CNSV	331-332, 2147-2148	2-3, 580-581
CCG47847.1	GARSV	757-758, 1153-1154, 1407-1408, 1679-1680	548-549, 669-670, 986-987
AUD08127.1	RCNVA	1166-1167, 1692-1693	682-683
SCN13017.1	AILV	1716-1717	556-557
BAA00234.1	BRSV	1176-1177, 1702-1703	692-693
AAN72830.1	TBRV	1176-1177, 1702-1703	692-693, 1175-1176
QPD02150.1	GNVA	484-485, 780-781	310-311, 940-941,
AAQ73821.1	ArMV	293-294	656-657, 952-953, 1986-1987, 2103-2104
CCE67066.1	GDefV	293-294	953-954
AFM91094.1	GFLV-GHu	293-294	953-954
NP_619689.1	GFLV-F13	293-294	953-954
YP_009507921.1	MMMoV	648-649	1486-1487
APW29209.1	PCMoV	1757-1758, 2064-2065, 2261-2262	2-3, 483-484
AGY34703.1	MMLRaV	1800-1801, 1909-1910	259-260, 2012-2013
AAB03785.1	TRSV	1750-1751	334-335, 981-982, 1504-1505
AFR67086.1	AeRSV	1764-1765, 2024-2025	348-349, 995-996, 1519-1520
AGZ62579.1	PBRSV	1774-1775	356-357, 1005-1006, 1529-1530

[a]GenBank accession number of the RNA1-encoded polyprotein amino acid sequence.
[b]Acronym of nepoviruses.
[c]Amino acid residue positions of the WG motif.
[d]Amino acid residue positions of the GW motif.

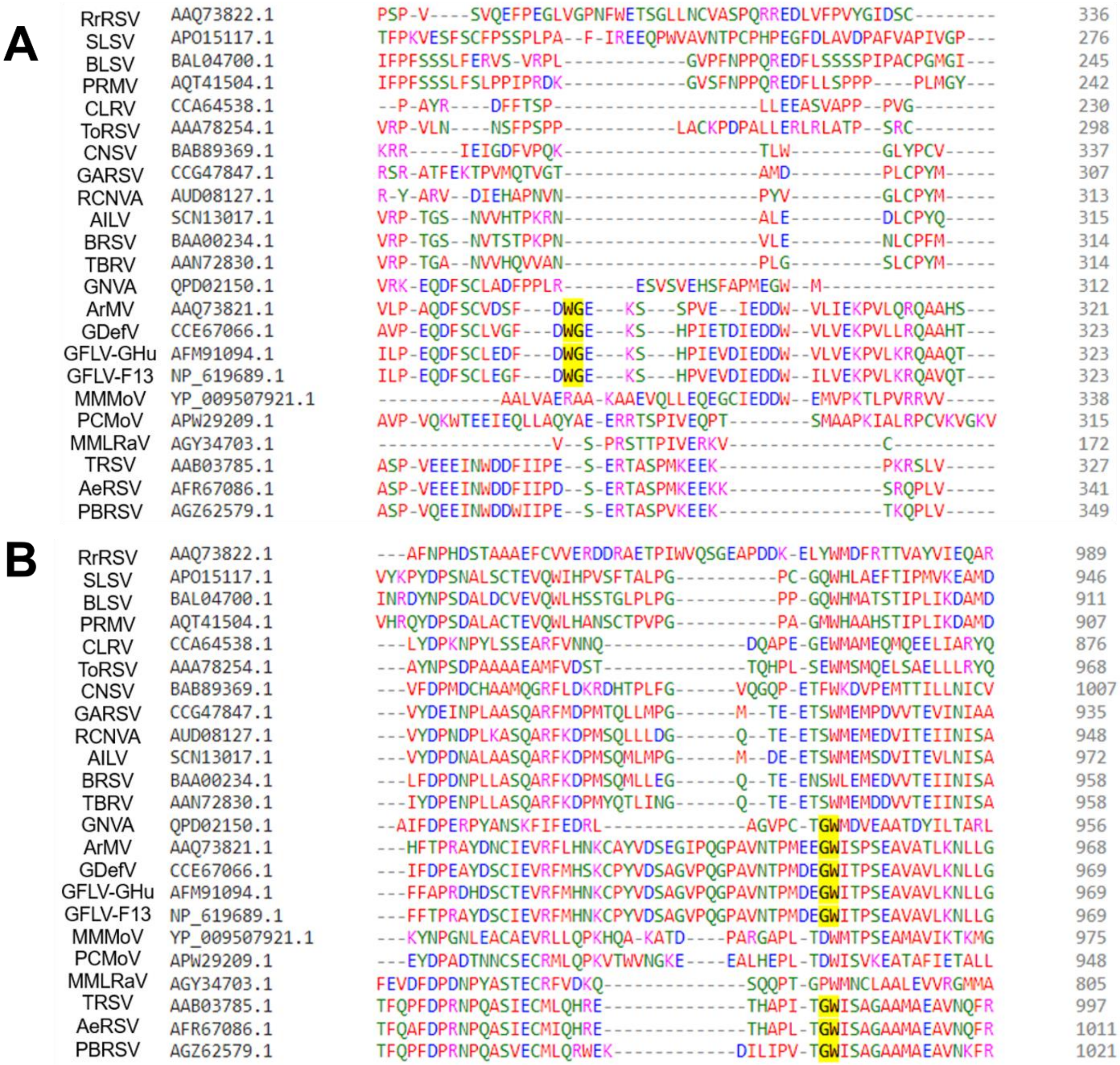

Supplementary Figure 1. Alignment of WG and GW of 22 nepovirus proteins 1A and 1B. (A) Multiple amino acid sequence alignments of nepovirus protein 1A using Clustal Omega for the presence of WG in yellow. (B) Multiple amino acid sequence alignments of nepovirus protein 1B using Clustal Omega for the presence of GW in yellow.

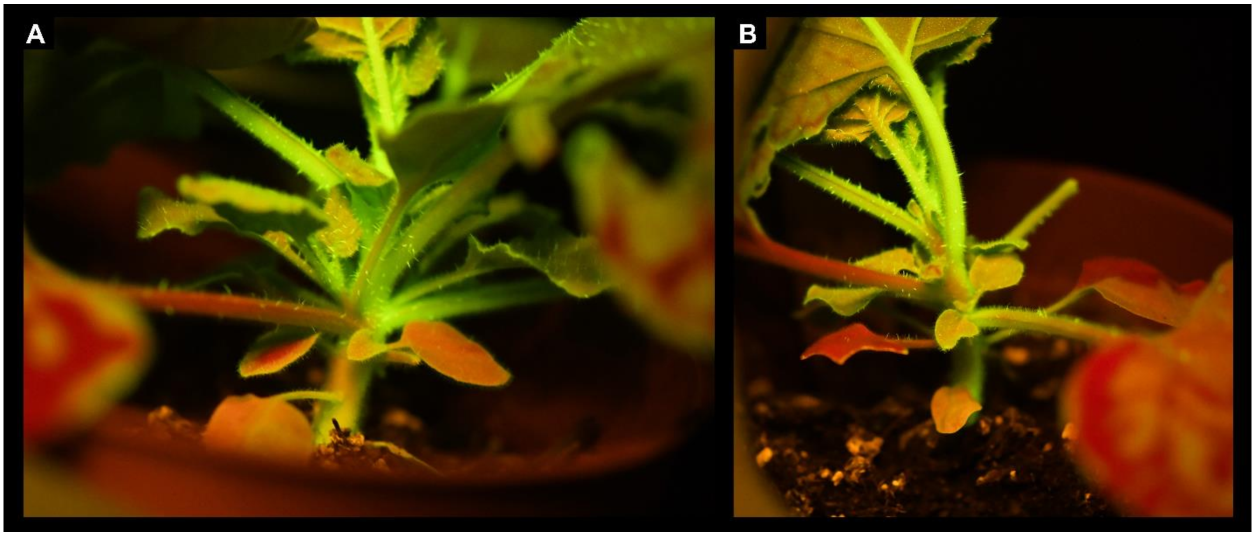

Supplementary Figure 2. Fluorescence images of stems of *Nicotiana benthamiana* 16c plants treated with pHELLSGATE8-EGFP and grapevine fanleaf virus (GFLV) protein 1B at 20 days post-infiltration. (A, B) Weak systemic RNA silencing is observed in the early phase of the infiltration but quickly fades away due to suppression by GFLV 1B as plants develop.

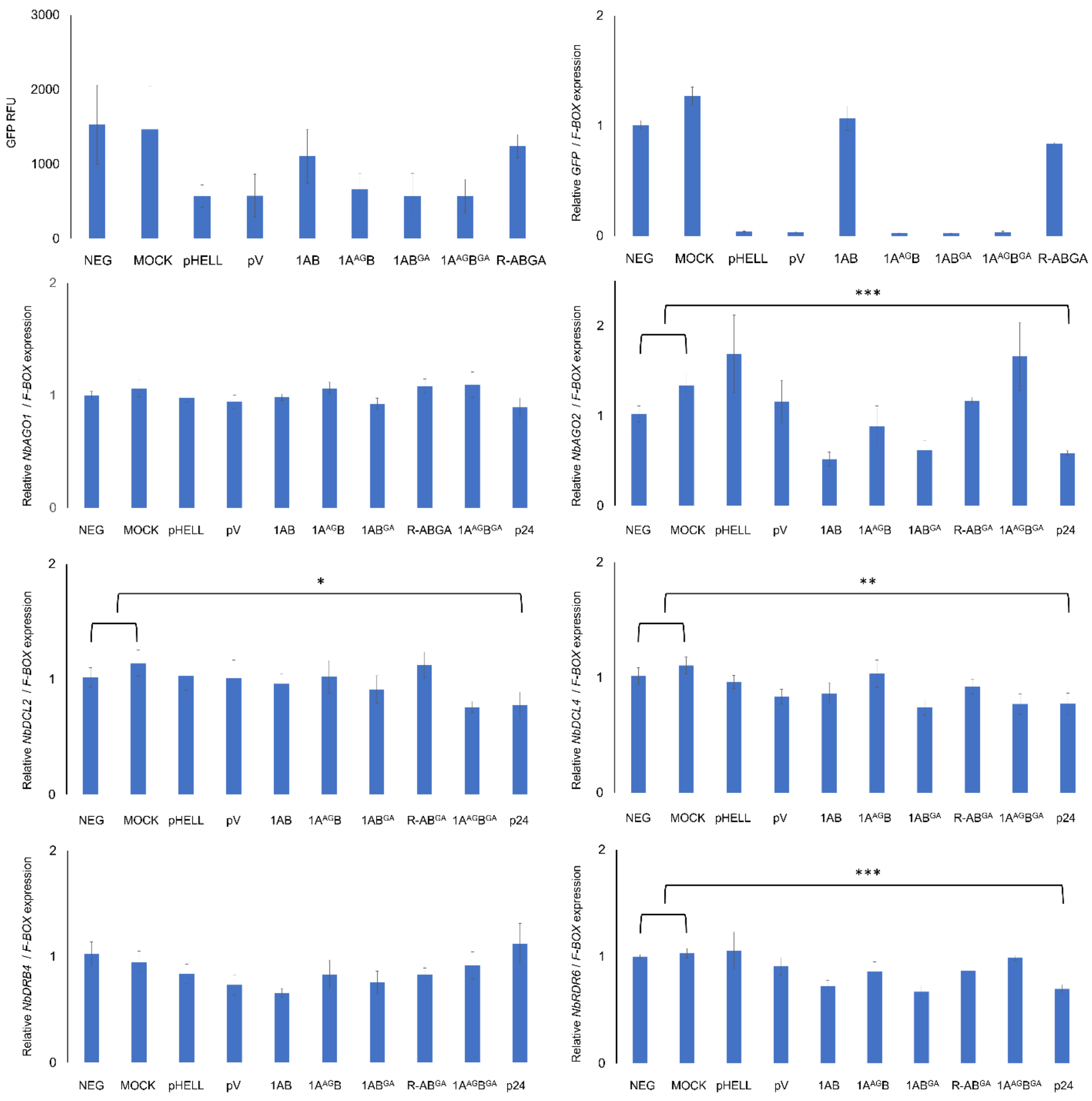

Supplementary Figure 3. Quantitative measurements of *GFP* expression in *Nicotiana benthamiana* 16c plants that developed systemic suppression upon co-infiltration with RNA silencing-inducing construct (pHELL) and grapevine fanleaf virus (GFLV) $1AB^{GA}$. Such plants are labeled as R-$1AB^{GA}$. GFP fluorescence intensity is exhibited in relative fluorescene units (RFUs). Relative *GFP* and host genes abundances normalized against host *F-BOX* and calibrated against untreated (NEG) plants are displayed. Three technical replicates were used per biological replicate (plant). Relative changes in host gene expressions by GFLV 1AB and its mutants and positive control GLRaV2 p24 are presented. Bars represent significant different between marked groups, and the asterisks denote significant differences ($P < 0.05$*, $P < 0.01$**, and $P < 0.001$***) compared with the

average value of untreated and mock-treated plants according to one-way ANOVA followed by Dunnett's post hoc multiple comparison test.

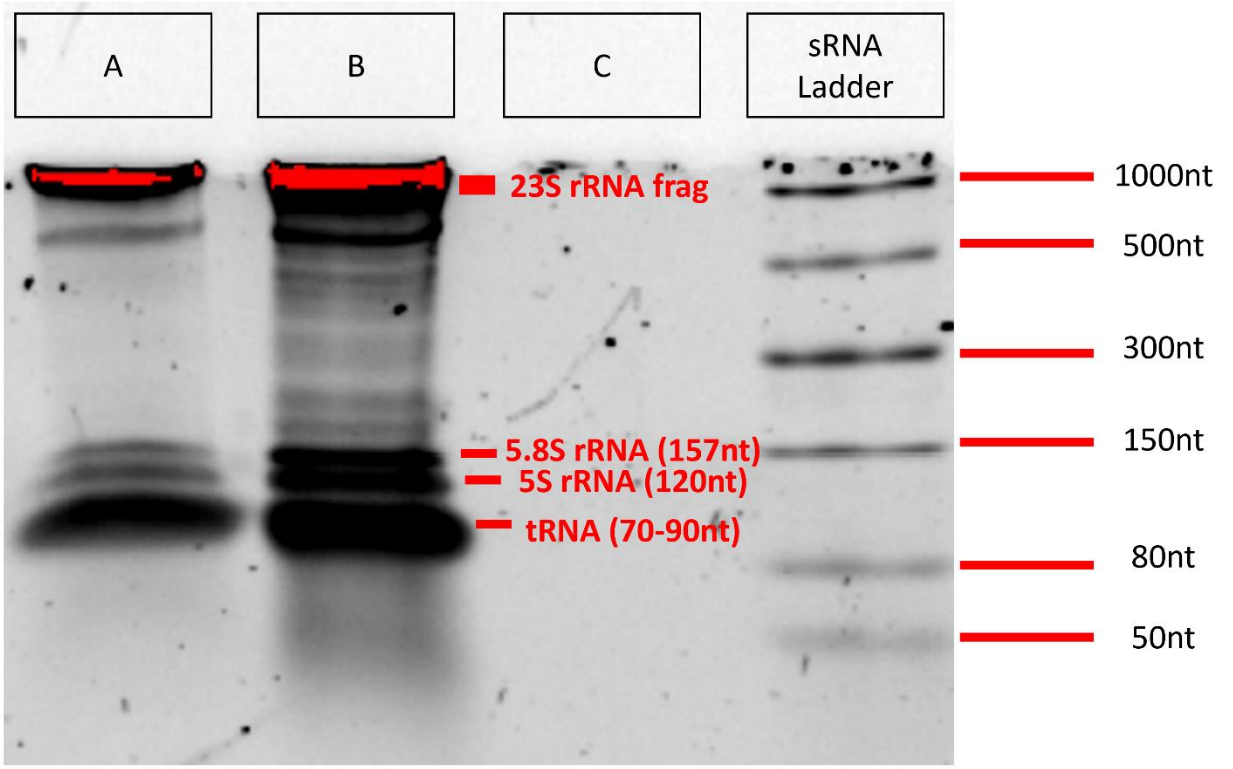

Supplementary Figure 4. Analysis of the integrity of small RNAs (sRNAs) by electrophoresis on a denaturing 15% polyacrylamide gel following staining with SYBR^{TM} Gold Nucleic Acid Gel Stain and visualization on a UV transilluminator. After mixing with Gel Loading Buffer II (Invitrogen) at 1:1 (v:v) ratio, (A) 200ng of sRNAs extracted from pV-treated *N. benthamiana* 16c plants, (B) 900ng of sRNAs isolated from 1B^{GA}-treated *N. benthamiana* 16c plants, and (C) no sRNA were loaded. A sRNA ladder (Low Range ssRNA Ladder, New England BioLabs) (right lane) was used to determine band size. Bands corresponding to ribosomal RNA (rRNA) and transfer RNA (RNA) are indicated. 23S rRNA fragment is abbreviated as 23S rRNA frag.

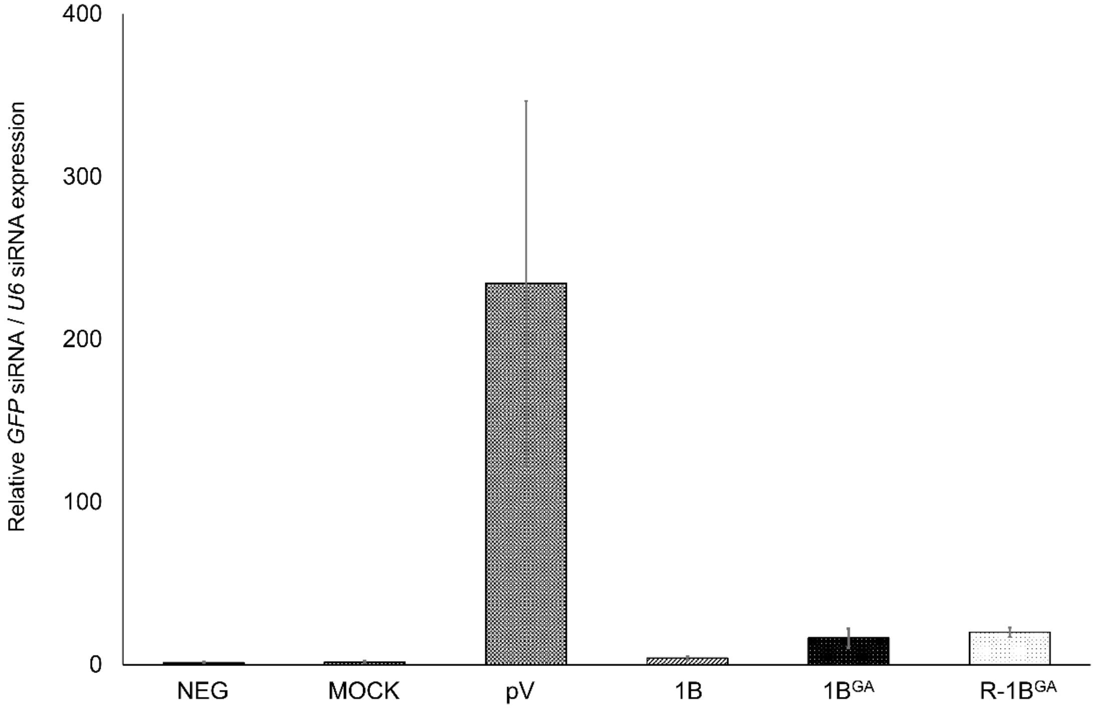

Supplementary Figure 5. Relative abundance of *GFP* siRNA accumulation in grapevine fanleaf virus 1B or 1B^{GA}-treated *Nicotiana benthamiana* 16c plants that developed RNA silencing systemically. With only two samples analyzed for R-1B^{GA}, no statistical analyses were performed.

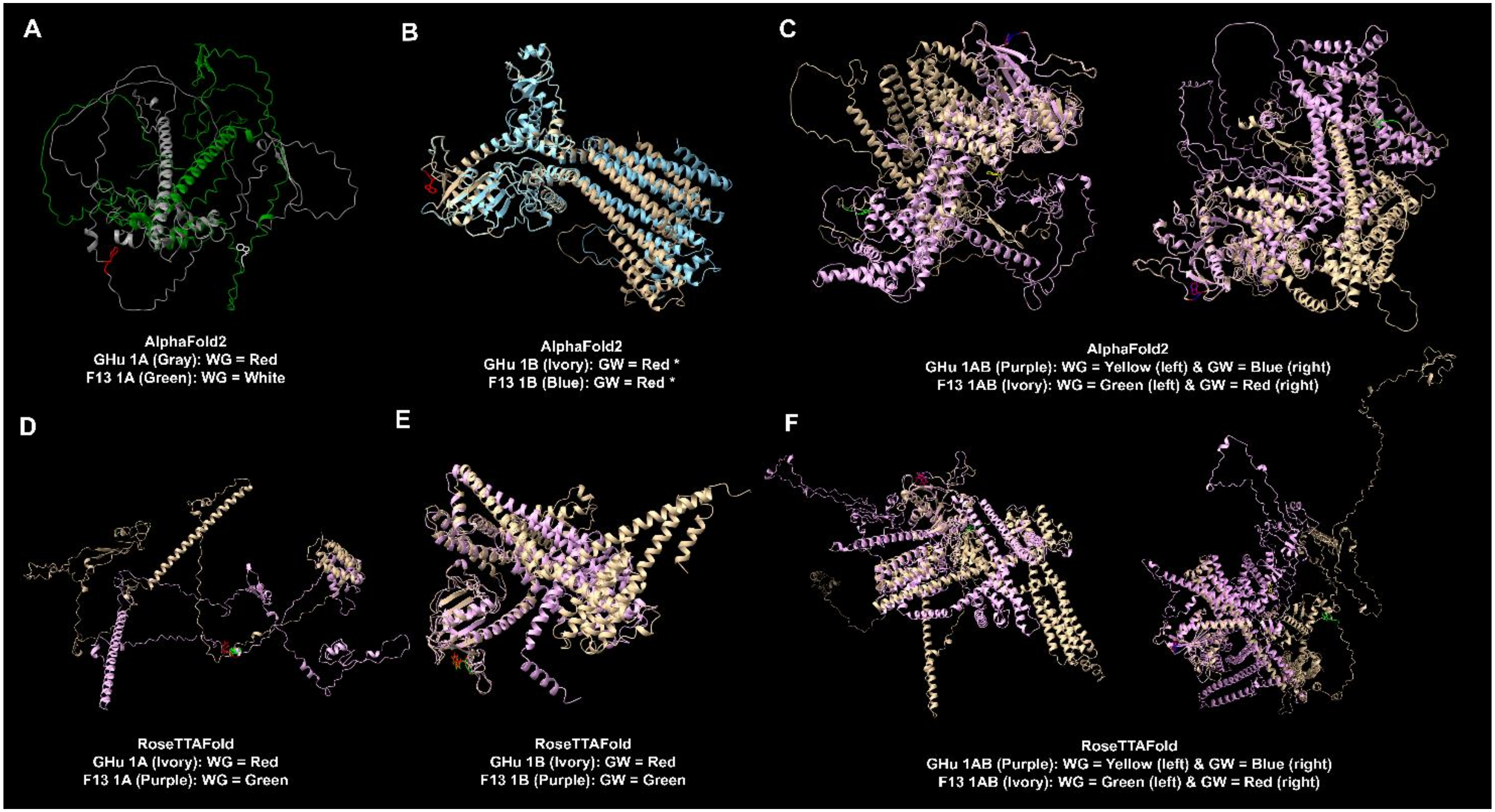

Supplementary Figure 6. Overlays of predicted structures of the suppressors of RNA silencing of grapevine fanleaf virus strains GHu and F13 obtained with AlphaFold2 (A, B, C) and RoseTTAFold (D, E, F) for proteins 1A (A, D), 1B (B, E) and 1AB (C, F). The WG/GW motifs are indicated in distinct colors as shown in the texts of the figure. The images were taken using ChimeraX.

A

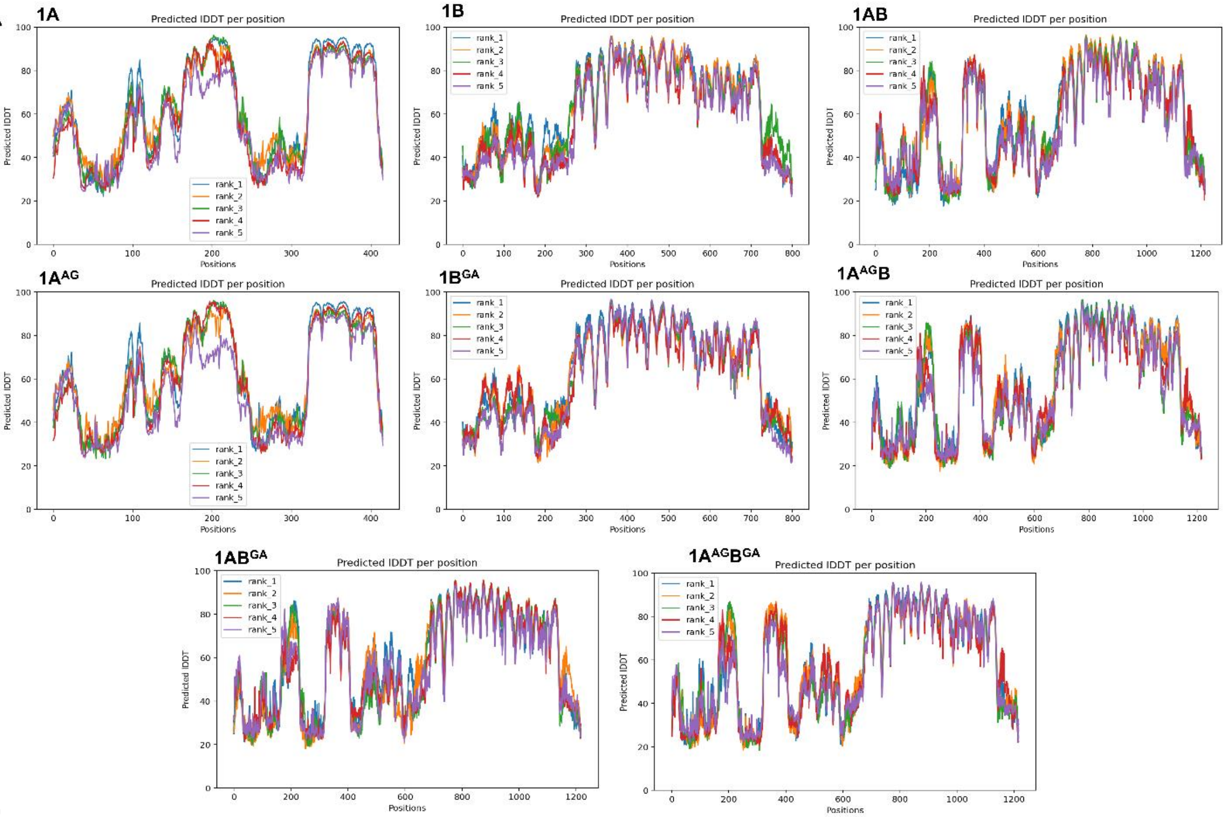

B

GFLV wildtype and mutant VSRs	RoseTTAFold confidence score[a]
1A	0.38
$1A^{AG}$	0.38
1B	0.62
$1B^{GA}$	0.59
1AB	0.40
$1A^{AG}B$	0.43
$1AB^{GA}$	0.40
$1A^{AG}B^{GA}$	0.41

Supplementary Figure 7. Confidence measurements of predicted protein structures of wildtype (1A, 1B, 1AB) and mutant ($1A^{AG}$, $1B^{GA}$, $1A^{AG}B$, $1AB^{GA}$, and $1A^{AG}B^{GA}$) suppressors of RNA silencing of grapevine fanleaf virus (GFLV) via AlphaFold2 and RoseTTAFold. (A) predicted local distance difference test (IDDT) scores from AlphaFold2 are displayed on the y-axis and their corresponding amino acid residues are shown on the x-axis. An IDDT score of 0 is the lowest confidence, and an IDDT score of 1 is the highest confidence for a model. Each line with different color indicates differently ranked models with rank 1 model having the highest confidence in prediction. (B) IDDT score via DeepAccNet showing protein structure prediction confidence from RoseTTAFold. A score of 0 implies a low confidence, while score of 1 implies a high confidence in protein structure prediction.

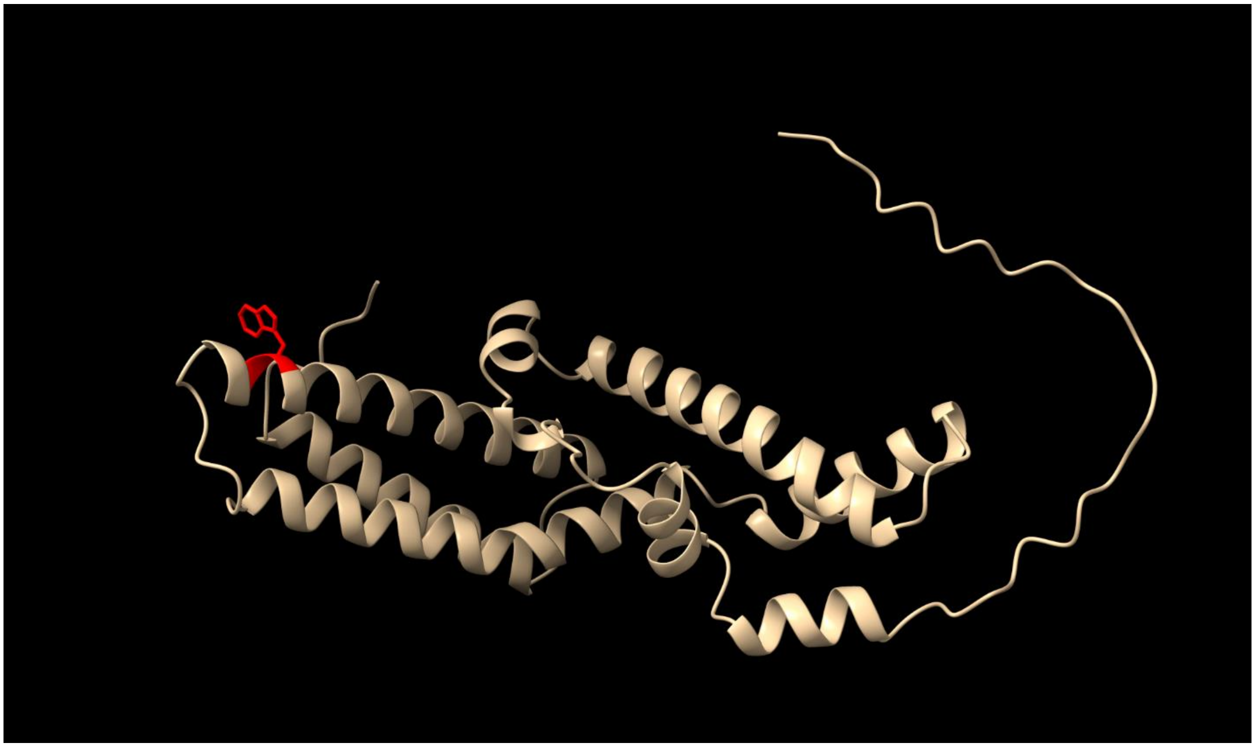

Supplementary Figure 8. Predicted structure of p24 encoded by grapevine leafroll-associated virus 2 isolate 14G466 (GenBank accession QBZ78614) obtained with AlphaFold2. The WG motif is shown in red. The image was taken using ChimeraX.

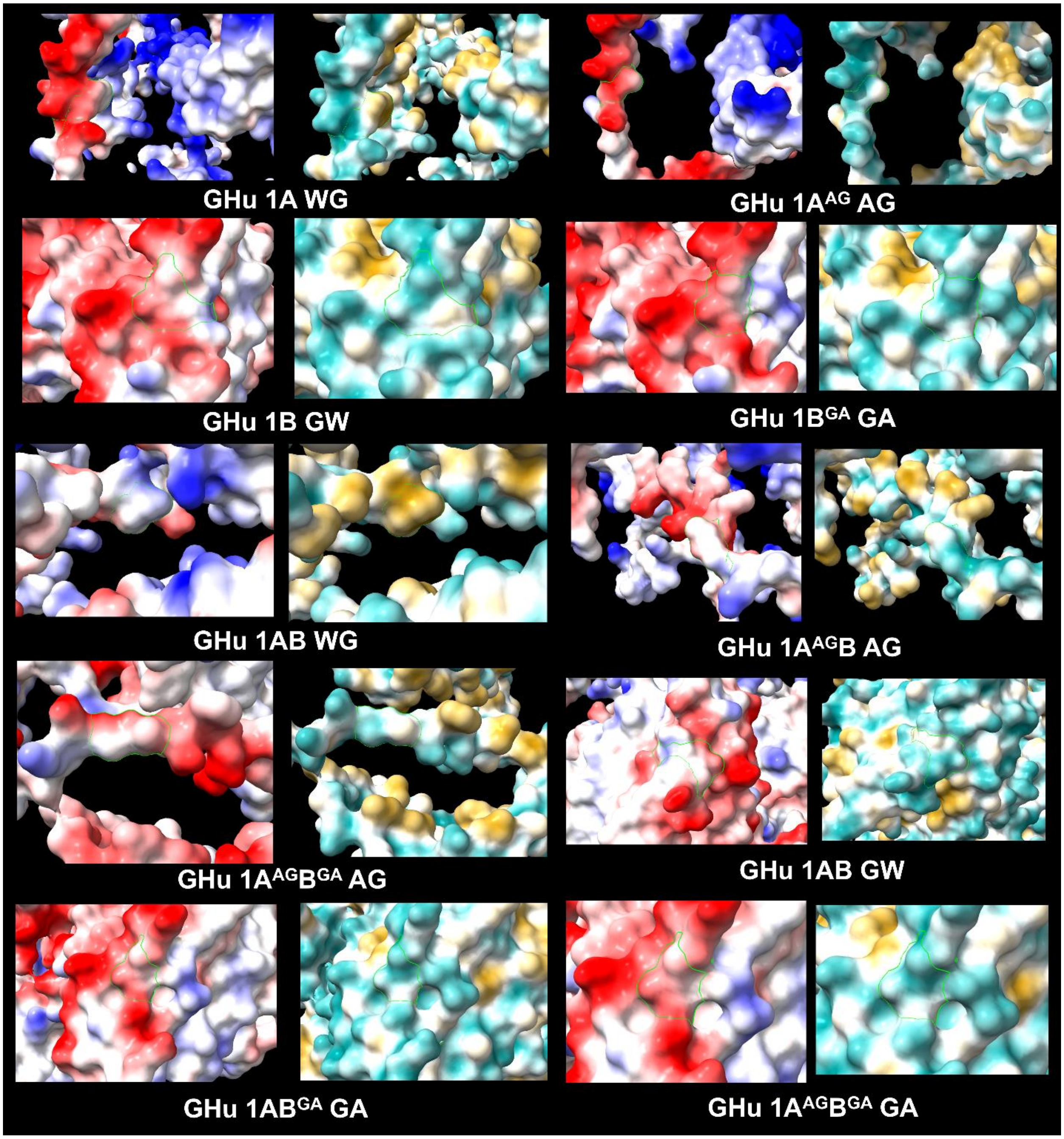

Supplementary Figure 9. The predicted chemical and physical properties of surrounding amino acid residues of the WG and GW motif structures in grapevine fanleaf virus (GFLV) wildtype (1A, 1B, and 1AB) silencing suppressors (VSRs) and their mutants ($1A^{AG}$, $1B^{GA}$, $1A^{AG}B$, $1AB^{GA}$, and $1A^{AG}B^{GA}$). Protein structures were predicted via AlphaFold2 and analyzed via ChimeraX with the structural locations of wildtype or mutated WG and GW motifs shown in green borderlines. The hydrophobicity of predicted protein structures is shown with cyan representing a hydrophilic surface, white representing a weak lipophilic surface, and gold representing a most lipophilic surface. The electrostaticity of predicted protein structures is shown with blue representing a positively charged surface, white representing a surface with neutral charge, and red representing a negatively charged surface. Images were captured by ChimeraX.
